# Transcriptomic characterization of human pancreatic CD206- and CD206 + macrophages

**DOI:** 10.1038/s41598-025-96313-y

**Published:** 2025-04-08

**Authors:** Alexander Jonsson, Olle Korsgren, Anders Hedin

**Affiliations:** 1 Department of Immunology, Genetics and Pathology, Uppsala University, Uppsala, Sweden; 2 Institute of Medicine, University of Gothenburg, Gothenburg, Sweden

**Keywords:** Human pancreas, Pancreatic macrophages, Diabetes, Transcriptomics, Pancreatic Islets, Diabetes, Pre-diabetes, Monocytes and macrophages, RNA sequencing, Islets of Langerhans

## Abstract

Macrophages reside in all organs and participate in homeostatic- and immune regulative processes. Little is known about pancreatic macrophage gene expression. In the present study, global gene expression was characterized in human pancreatic macrophage subpopulations. CD206- and CD206 + macrophages were sorted separately from pancreatic islets and exocrine tissue to high purity using flow cytometry, followed by RNA-seq analysis. Comparing CD206- with CD206 + macrophages, CD206- showed enrichment in histones, proliferation and cell cycle regulation, glycolysis and SPP1-associated immunosuppressive polarization while CD206 + showed enrichment in complement and coagulation-, IL-10 and IL-2RA immune regulation, as well as scavenging-related gene sets. Comparing islet CD206- with exocrine CD206-, enrichments in islet samples included two sets involved in immune regulation, while enrichments in exocrine samples included sets related to extracellular matrix and immune activation. Fewer differences were found between CD206 + macrophages, with enrichments in islet samples including two IL2-RA related gene sets, while enrichments in exocrine samples included sets related to extracellular matrix and immune activation. Comparing macrophages between individuals with normoglycemia, elevated HbA1c or type 2 diabetes, only a few diverse differentially expressed genes were identified. This work characterizes global gene expression and identifies differences between CD206- and CD206 + macrophage populations within the human pancreas.

**Supplementary Information:**

The online version contains supplementary material available at 10.1038/s41598-025-96313-y.

## Introduction

Macrophages are innate immunity effector cells with phagocytic, microbiocidal and antigen-presenting capabilities that also have a role in tissue remodeling and clearance of cellular debris^1^. Macrophages are heterogeneous and can be subdivided in several ways. Plenty of literature has used the M1 and M2 subdivision originally reported in in vitro experiments^2^. Importantly, macrophages are highly plastic cells and the M1 and M2 (and similar classifications) may reflect in vitro-induced extremes of macrophage phenotypes^3^. Nevertheless, they can be useful as a way to conceptualize macrophage function under specific conditions. As typically described, classically activated (M1) macrophages have enhanced microbiocidal capability and secrete pro-inflammatory cytokines that enhance Th1 activity^4^. Alternatively activated (M2) macrophages have enhanced phagocytic and scavenging capabilities^4^ and secrete cytokines that promote immunosuppression, tissue remodeling and Th2 effector responses^5^.

Expression of CD206 is regulated by various cytokines and expression of CD206 is considered a marker of an M2-like macrophage phenotype^6^. Pancreatic infiltration of CD206 + macrophages is increased in adult organ donors with a longer stay in the intensive care unit, and increased infiltration correlates with an increase in the proliferation of beta cells and other parenchymal cells^7^. Similarly, CD206 + macrophage infiltration is essential for beta-cell proliferation in mice following experimentally induced injury through partial duct ligation^8^. Early depletion of macrophages in the op/op mouse leads to decreased beta cell mass in fetuses and adults^9^, and macrophages are present in human islets of Langerhans during embryogenesis^10^, suggesting their involvement also in human islet development. Thus, macrophages are likely to be involved in pancreatic endocrine cell proliferation and development.

Macrophages are also involved in several pancreatic diseases. There is various evidence implicating macrophages in the development of diabetes^11–14^. In pancreatic ductal adenocarcinoma, tumor-associated macrophages are involved in immunosuppression, angiogenesis, matrix remodeling and metastasis^15^, and higher macrophage density is associated with worse prognosis^16^. In acute pancreatitis, pancreatic macrophages contribute to local inflammation and trypsinogen activation, while in chronic pancreatitis interactions between M2 macrophages and pancreatic stellate cells contribute to fibrosis^17^.

In summary, previous findings suggest involvement of macrophages in pancreatic tissue repair, islet development and proliferation, as well as in disease processes affecting the pancreas. Little is known about pancreatic macrophage gene expression, both in subjects with no known pancreatic disease and in diabetes. In the present study, we characterized global gene expression in primary human pancreatic macrophages from organ donors using RNA-seq. Macrophages were sorted from separate islet- and exocrine tissue suspensions and further subdivided by their expression of CD206 due to previous use of this marker in a study involving pancreatic macrophages^7^. Transcriptomes were then compared between pancreatic islet- and exocrine CD206- and CD206 + macrophage populations, and between donors with normal HbA1c, intermediately elevated HbA1c and type 2 diabetes.

## Results

### Macrophage cell sorting purity and CD206 + frequency prior to cell sorting

**Fig. 1:**
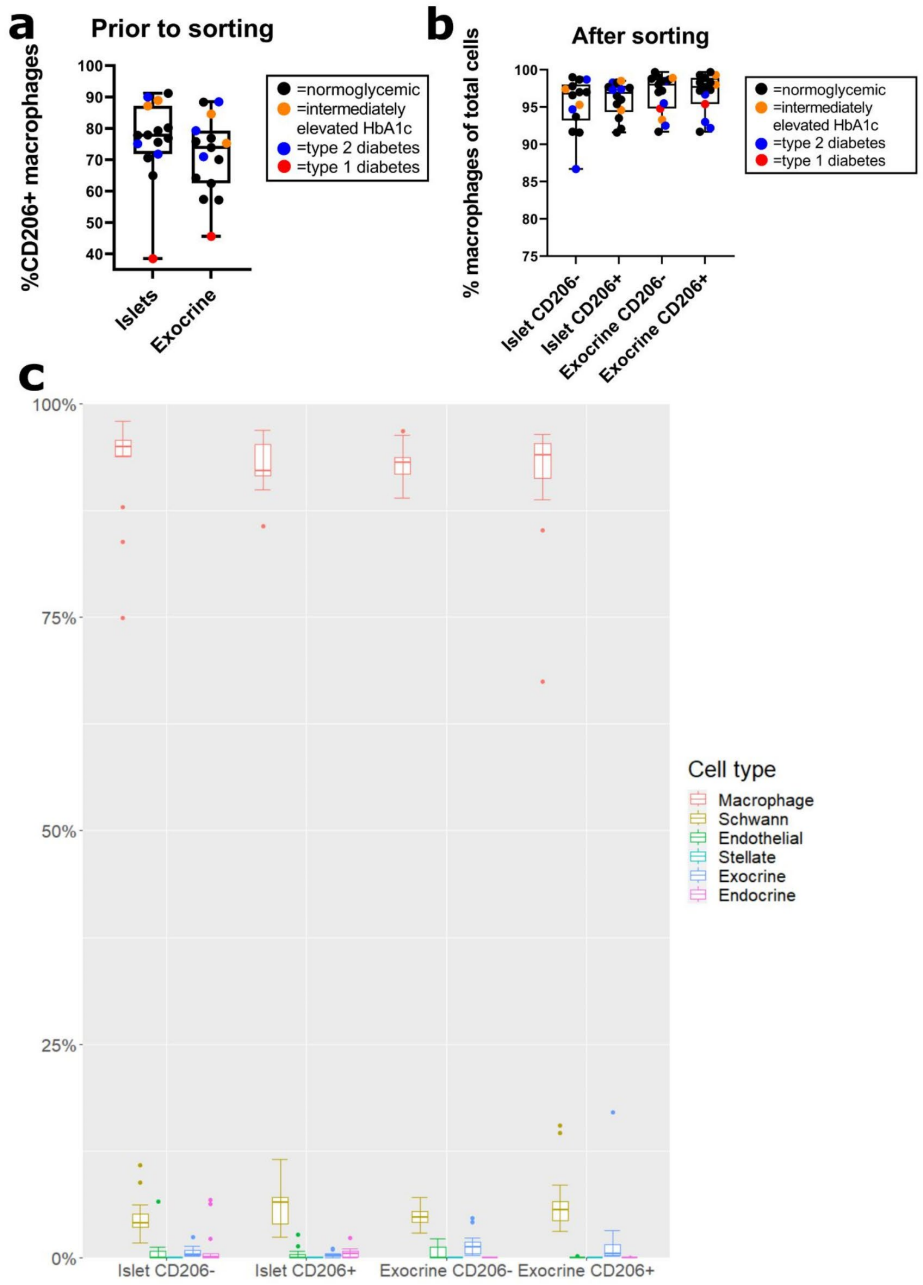
CD206 + frequency prior to cell sorting and macrophage purity after cell sorting. **a** In FACS-analysis of macrophages prior to sorting, the fraction of macrophages expressing CD206 was similar in islet- and exocrine samples (median 77.8 and 73.9% respectively). The majority of macrophages were CD206 + in all donors except the donor with T1D (highlighted in red). Donors with impaired glucose metabolism or type 2 diabetes are highlighted in orange and blue. **b** In FACS analysis after cell sorting, the median post-sorting macrophage purity (number of CD45 + HLA-DR + C64 + cells/number of total cells) was similar in all subgroups (96.8%, 96.9%, 98.0% and 97.7% for islet CD206-, islet CD206+, exocrine CD206- and exocrine CD206 + samples respectively). **c** Post-sorting purity was evaluated in the bulk transcriptomic data using multi-subject single cell deconvolution (MuSiC), with similar macrophage purities in all subgroups (95%, 92.1%, 93.1% and 94% respectively).

Prior to sorting, the majority of macrophages were CD206 + in all samples except the samples from the donor with T1D. As determined by FACS, the median fraction of CD206 + cells was 77.8% and 73.9% among islet and exocrine macrophages respectively (Fig. 1a). In the donor with T1D, the fractions of CD206 + macrophages were substantially lower with a frequency of 38.5% in islets and 45.6% in exocrine tissue. Median post-sorting macrophage purity was 96.8% according to FACS (Fig. 1b) and 93.5% according to MuSiC-analysis (Fig. 1c). Post-sorting macrophage purities were similar in all subgroups. After sorting, CD206- samples frequently contained a small fraction of slightly CD206 positive macrophages and vice versa – the median fraction of macrophages with correct CD206 expression after sorting was 98.2% (supplementary figure S1).

### FACS analysis of HLA-DR expression

The CD206 + macrophages had a higher expression of HLA-DR than the CD206- macrophages at the protein level as determined by mean fluorescence intensity (supplementary figure S2).

### Correlations between donor characteristics and macrophage content and phenotype

Correlations between donor characteristics (age, BMI, HbA1c, duration of ICU stay, cold ischemia time, glucose-stimulated insulin secretion and sex), macrophage content, and fraction of CD206 + macrophages were examined visually and using Spearman’s rank coefficient or Mann Whitney test (Supplementary figure S3). Statistically significant correlations were found between HbA1c and macrophage content in exocrine samples and fraction of CD206 + macrophages in islets and exocrine samples. A significant correlation was also found between BMI and fraction of CD206 + macrophages in exocrine samples. These analyses were not corrected for multiplicity.

### Principal component analysis of sorted macrophages and analysis of M2, monocyte and neutrophil marker gene expression

**Fig. 2:**
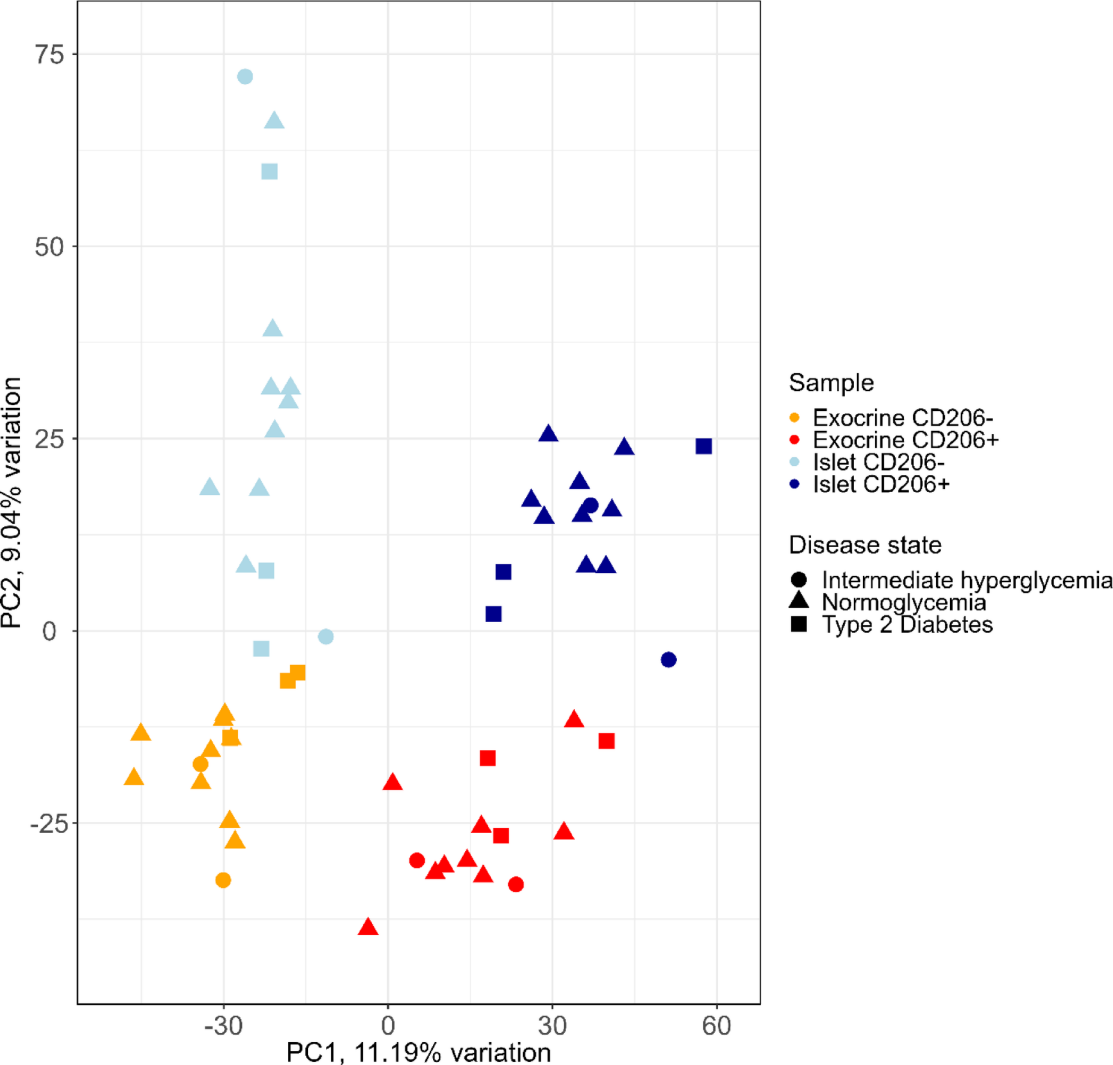
Principal component analysis of sorted pancreatic macrophage samples. Four sorted macrophage samples per donor were analyzed from fifteen organ donor pancreases. Sample subgroup is shown by color and disease status is shown by symbol. Macrophage phenotypes (CD206- and CD206+) clustered separately across PC1. Exocrine- and islet sample origin clustered separately across PC2. No clustering pattern was evident for disease states.

Principal component analysis indicated clustering by macrophage phenotype on principal component 1, where 11.19% of the variation was explained, and by tissue origin on principal component 2, where 9.04% of the variation was explained (Fig. 2). The samples clustered by sorting subgroup. There was no evident clustering by disease status.

As an initial quality control step, the markers used in cell sorting, M2 macrophage markers, monocyte markers and neutrophil markers were analyzed in the differential gene expression lists between islet CD206- and CD206 + samples as well as exocrine CD206- and CD206 + samples. The following paragraph describes the results of these gene subset-analyses.

Gene expression of the macrophage sorting markers (*PTPRC*, *FCGR1A* and *HLA-DRA*) did not differ between sample groups and were also assessed by plotting CPM (Supplementary Figure S4). As expected, *MRC1* (CD206) expression was enriched in CD206 + samples in both islets and exocrine tissue (Supplementary Figure S4). The expression of the commonly used intracellular macrophage marker *CD68* was higher in CD206 + samples (Supplementary Figure S4). The difference in both of these genes were statistically significant in the differential gene expression analyses.

There was a higher expression in CD206 + macrophages of many previously reported M2-markers (Supplementary Table S1), such as *CD163* and *MSR1*, *CCL18*, *CD209*, *CLEC10A*, *IGF1*, *IL10* and *MERTK*. No differences were identified for other markers such as *CD86*, *TGFB1*, *NOS2*, *ARG1*, *CLEC7A* or *VEGFA*. *IL1RN* was enriched in islet CD206 + but not in exocrine CD206+.

For the 67 canonical monocyte markers analyzed, 17 were differentially expressed in comparisons between exocrine CD206- and CD206 + macrophages and 21 were differentially expressed in comparisons between islet CD206- and CD206 + macrophages (Supplementary Table S2). The differentially expressed monocyte markers were similarly split between CD206- and CD206 + in both tissues (9 vs. 8 and 10 vs. 11 respectively).

For the 77 neutrophil-associated genes, 18 were differentially expressed in comparisons between exocrine CD206- and CD206 + samples and 23 were differentially expressed in comparisons between islet CD206- and CD206 + samples (Supplementary Table S3). Enrichment was more frequent in CD206- samples than in CD206 + samples (13 vs. 5 and 15 vs. 8 for exocrine and islet samples respectively). All of the differentially expressed neutrophil markers are also expressed in macrophages according to single-cell RNA expression data in the human protein atlas database.

### Differential gene expression in CD206- and CD206 + macrophages in Islets and exocrine pancreas

**Fig. 3:**
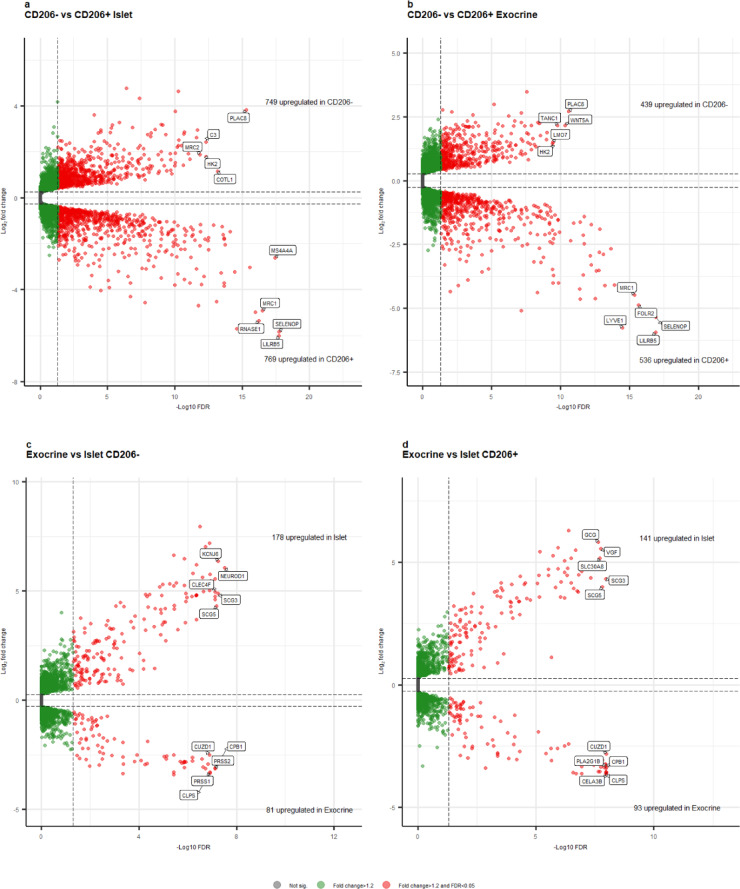
Differential gene expression in pancreatic macrophage subgroups. Differential gene expression analysis was performed pairwise between: **a** islet CD206- and islet CD206+, **b** exocrine CD206- and exocrine CD206+, **c** exocrine CD206- and islet CD206-, and **d** exocrine CD206 + and islet CD206+. The significantly differentially expressed genes (fold-change > 1.2 and FDR < 0.05) are depicted by red circles, and the top 5 differentially expressed genes are highlighted in each comparison.

In islets, 1,518 genes were differentially expressed between CD206- and CD206 + macrophages, with higher expression of 749 genes in CD206- macrophages and higher expression of 769 genes in CD206 + macrophages (Fig. 3a).

In exocrine tissue, 975 genes were differentially expressed between CD206- and CD206 + macrophages, with higher expression of 439 genes in CD206- macrophages and 536 genes in CD206 + macrophages (Fig. 3b).

Comparing CD206- macrophages between islets and exocrine tissue, 259 genes were differentially expressed, with 178 enriched genes in islet samples and 81 enriched genes in exocrine samples (Fig. 3c).

Comparing CD206 + macrophages between islets and exocrine tissue, 234 genes were differentially expressed, with 141 enriched genes in islet samples and 93 enriched genes in exocrine samples (Fig. 3d).

In these comparisons of CD206- samples and CD206 + samples between tissues, several parenchymal cell expressed genes (such as *INS*, *AMY2A*) were differentially expressed.

Full tables of differentially expressed genes are provided in the supplementary materials (Supplementary Table S4-S7).

### Gene set enrichment-comparisons between pancreatic CD206- and CD206 + macrophages

**Fig. 4:**
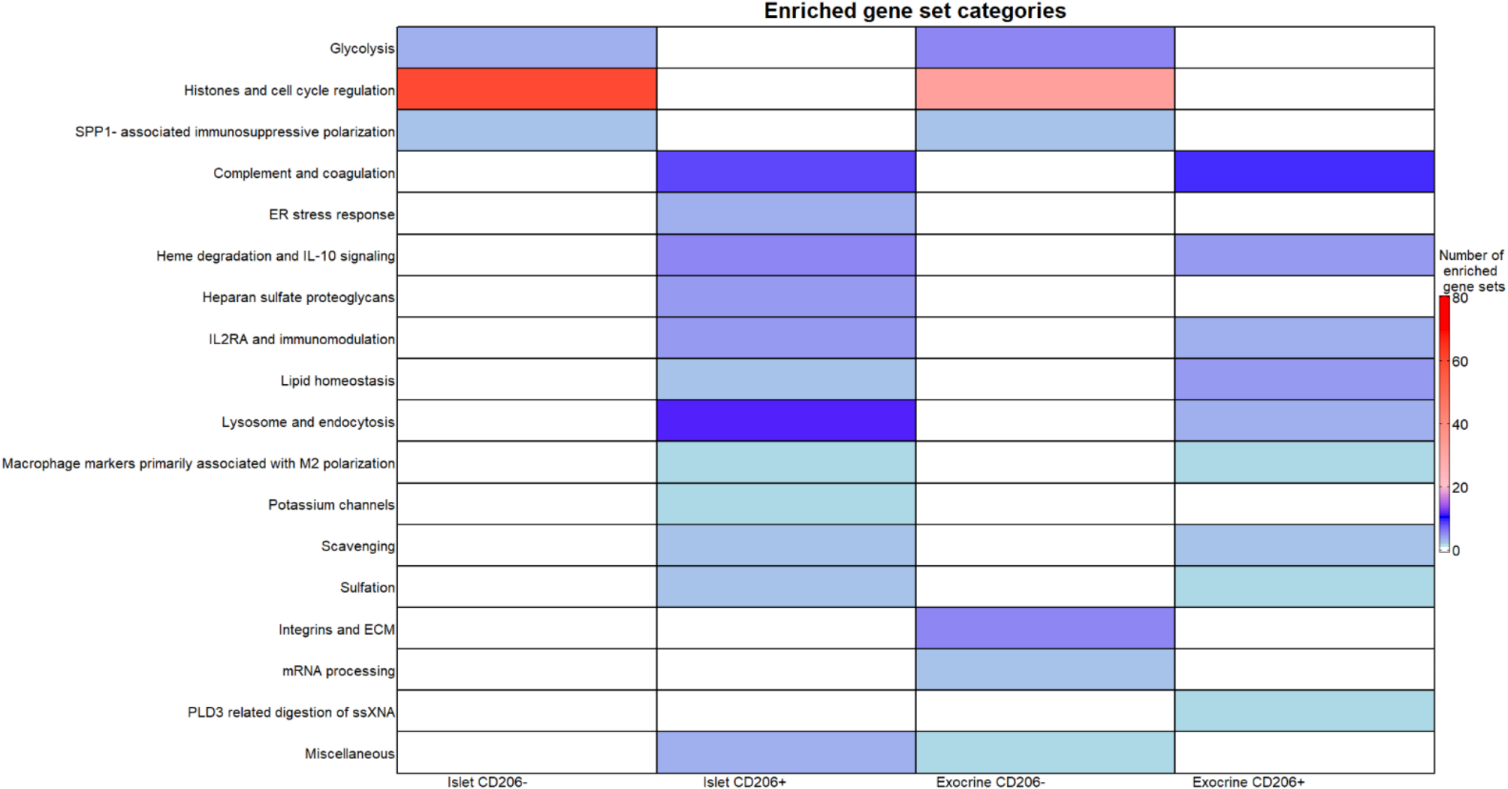
Gene set enrichment summary for within-tissue analyses. Enriched gene sets were manually annotated into categories to facilitate interpretation. The number of significantly enriched gene sets (for the pairwise analyses comparing CD206- with CD206 + samples within islets and exocrine tissue respectively) in each category are displayed in a heatmap. In comparisons between islet macrophages, for CD206- most sets were annotated to “histones and cell cycle regulation” while for CD206 + most sets were annotated to “complement and coagulation”. In comparisons between exocrine macrophages, for CD206- most sets were annotated to “histones and cell cycle regulation” while for CD206 + most sets were annotated to “complement and coagulation”.

CAMERA analysis in islets identified significant differences in 111 gene sets, with 65 enriched sets in islet CD206- macrophages and 46 enriched sets in islet CD206 + macrophages. The enriched sets in islet CD206- macrophages could broadly be classified into histones and cell cycle regulation, SPP1-associated immunosuppressive polarization and glycolysis (Fig. 4). Most of the enriched sets in islet CD206 + macrophages could broadly be classified into complement and coagulation, heme degradation and IL-10 signaling, lipid homeostasis, macrophage markers primarily associated with M2 polarization, IL2RA and immunomodulation, sulfation, scavenging, lysosome and endocytosis, heparan sulfate proteoglycans, ER stress response and potassium channels (Fig. 4). The gene sets are additionally listed in Supplementary Table S8.

CAMERA analysis in exocrine tissue (Fig. 4) identified significant differences in 75 gene sets, with 47 enriched sets in CD206- and 28 enriched sets in CD206 + macrophages. The 47 enriched sets in exocrine CD206- macrophages could broadly be classified into histones and cell cycle regulation, SPP1- associated immunosuppressive polarization, glycolysis, integrins and ECM, and mRNA processing (Fig. 4).

The enriched sets in exocrine CD206 + macrophages could broadly be classified into complement and coagulation, heme degradation and IL-10 signaling, lipid homeostasis, macrophage markers primarily associated with M2 polarization, IL2RA and T cell inhibition, sulfation, scavenging, lysosome and endocytosis and PLD3 related digestion of ssXNA (Fig. 4). The gene sets are additionally listed in Supplementary Table S9.

The overlapping genes between gene set categories is displayed in Supplementary Figure S5-S8.

### Gene-set enrichment comparisons between islet and exocrine macrophages

Functional enrichment analysis between CD206- samples identified enrichment in 31 gene sets in islet CD206- and 150 gene sets in exocrine CD206- macrophages (Supplementary Table S10). Seemingly, the majority of enriched sets (such as REACTOME_REGULATION_OF_GENE_EXPRESSION_IN_BETA_CELLS, REACTOME_DIGESTION) in this comparison were related to endocrine or exocrine functions or general cellular processes. In islet CD206- samples, two of the enriched sets (REACTOME_PD_1_SIGNALING and REACTOME_BUTYROPHILIN_BTN_FAMILY_INTERACTIONS) seemed less likely to be involved in endocrine functions. In exocrine CD206- samples, 32 of the enriched sets seemed less likely to be explained by exocrine cell functions. These sets were related to extracellular matrix regulation, iron metabolism, immune function, antigen presentation, antimicrobial peptides/defensins, and cell surface interactions at the vascular wall.

Functional enrichment analysis between CD206 + samples identified enrichment in 37 gene sets in islet CD206 + and 69 gene sets in exocrine CD206 + macrophages (Supplementary Table S11). Similar to the comparison between CD206-, the majority of sets were related to parenchymal cell functions or general cellular processes.

In islet CD206+, three of the enriched sets had more apparent relevance in macrophages (PID_IL12_STAT4_PATHWAY, PID_TCR_CALCIUM_PATHWAY, and WP_MIRNA_TARGETS_IN_ECM_AND_MEMBRANE_RECEPTORS).

In exocrine CD206+, eight of the enriched sets had more apparent relevance in macrophages. These sets were involved in processes related to alpha-defensins, extracellular matrix, and immune activation and all but one (REACTOME_SIGNALING_BY_ROBO_RECEPTORS) were also enriched in exocrine CD206- compared with islet CD206-.

### Differential gene expression and gene set enrichment comparisons between macrophages from normoglycemic donors and donors with intermediate hyperglycemia or type 2 diabetes

Comparisons of the macrophage subgroups between normoglycemic donors and donors with intermediately elevated HbA1c found significant differences only between islet CD206- macrophages, with 4 differentially expressed genes (Supplementary Table S12).

Comparisons of the macrophage subgroups between normoglycemic donors and donors with type 2 diabetes found significant differences between islet CD206- macrophages (4 differentially expressed genes) (Supplementary Table S12) and between exocrine CD206 + macrophages (35 differentially expressed genes) (Supplementary Table S13).

No significant enrichments were identified through CAMERA.

## Discussion

Transcriptomic profiles were generated in bulk-sorted CD206- and CD206 + macrophages from human pancreatic islets and exocrine tissue immediately after islet isolation. Gene expression profiles were generated from highly purified pancreatic macrophages, as shown by flow cytometry and deconvolution analysis. The deconvolution analysis suggested that Schwann cells was the second most common cell type in all samples. This cell type was very uncommon (0.02%) in the original data set (Tosti) and most likely reflects some technical artefact. There was no evident bias between CD206- and CD206 + in the enrichment of monocyte-associated markers, supporting similar macrophage purities. The use of primary freshly isolated human pancreatic macrophages from pancreases procured for organ transplantation and sorted at a high purity was a strength of this study. In rat islets, HLA class 2 + cells and leukocytes have previously been shown to decrease in frequency after 4 days of culture^18^.

CD206 has recently been used as a macrophage subset marker in the intestine^19^ and has been suggested as a marker of phagocytic macrophage subpopulations in murine bone marrow and intestine^20^. In principal component analysis, our samples clustered by CD206 expression along PC1. Additionally, multiple genes previously associated with the M2 phenotype were enriched in the CD206 + samples in both tissues. Thus, our findings indicate that CD206 is a relevant marker of an M2 macrophage-like phenotype within the human pancreas. Further, prior to sorting the majority of macrophages were CD206+, with similar proportions in islet- and exocrine tissue isolates. While islet isolation and single-cell processing could affect the pre-sorting CD206 + proportions, the majority of intra-islet macrophages expressing CD206 is also in line with a previous organ donor tissue section-based study in which CD68 + CD206 + cells constituted 73.4% of all CD68 + cells in pancreatic tissue^7^. Intriguingly, while the expression of the cell surface marker HLA-DR did not differ at the RNA level, expression was higher in the CD206 + macrophages at the protein level. The donor with type 1 diabetes was the only donor in our study where the CD206- macrophage proportions were higher than the CD206 + proportions, but unfortunately the amount of available islet tissue was very low (100 islets instead of the 20,000 islet equivalents available for the other donors) and the number of obtained islet macrophages was too low to allow analysis of their transcriptomics.

Comparisons between the CD206- and CD206 + macrophages within islets and exocrine tissue identified a large number of differentially expressed gene sets and genes, with similar gene set-level differences.

CD206- macrophages had enrichment in gene sets related to proliferation and the cell cycle, histones, and glycolysis. This could indicate a higher degree of proliferation in the CD206- macrophages compared with CD206 + macrophages. However, in response to LPS macrophages may release microvesicles coated with histones that stimulate inflammation^21^, potentially linking this finding to inflammation. M1 macrophages are heavily dependent on glycolysis for energy production^22^, and the enrichment in glycolysis-related sets in our CD206- samples could reflect similar changes in energy metabolism.

CD206- macrophages also had enrichment in two gene sets involving SPP1. SPP1 has pleiotropic effects. It is important in cell-mediated immune responses and suppresses IL-10 production^23^. SPP1 improves the function of transplanted rat islets exposed to IL-1β and protects an insulin-producing cell line from IL-1β mediated cytotoxicity^24^. However, the role of CD206- macrophage expression of SPP1 in pancreatic immune balance and islet cell function remains to be clarified.

There were several enriched complement-associated sets in CD206 + macrophages. At the gene level, enrichment included complement factor genes *C2*, *C1QA*, *C1QB* and *C1QC*, complement receptors *CR1*, *C3AR1*, *VSIG4* and *C5AR1*, and complement regulators *CD46*, *CD59* and *SERPING1*. Three of these (*CD46*,* CD59*,* C3AR1*) were significantly enriched only in islet CD206 + macrophages. Interestingly, complement *C3* was enriched in CD206- macrophages in both tissues. Complement is involved in chemotaxis and stimulation of phagocytosis, and expression of complement components has been described in macrophages previously^25,26^. VSIG4 is involved in phagocytosis of C3-opsonized pathogens^27^ and expression has been previously reported in a subset of macrophages^28^. C1Q has been reported to stimulate macrophage efferocytosis activity through upregulation of MER and its ligand GAS6^29^, which were also enriched in the CD206 + samples in our study.

Further, CD206 + macrophages showed enrichment in heme degradation, lipid homeostasis, and scavenging pathways, consistent with scavenging functions.

Additionally, CD206 + samples showed enrichment in immunoregulatory pathways related to IL-10 and IL2-RA. IL-10 is a cytokine with anti-inflammatory effects through multiple mechanisms^30^. IL-10 receptor subunits are expressed in human alpha- and beta cells^31^ and IL-10 has been proposed to promote beta cell survival and function together with IL-4 and IL-13^32^. Increased levels of IL-10 have been shown to either increase or alleviate islet inflammation in the NOD-mouse model of diabetes depending on timing^30^.

IL2-RA is part of the heterotrimeric IL2-receptor and is expressed in T-cells and other immune cells^33^. Some polymorphisms in *IL2RA* are associated with increased risk of developing type 1 diabetes^34^. Higher expression of *IL2RA* is associated with worse outcomes in pancreatic ductal adenocarcinoma^35^. Thus, in our highly purified samples of CD206- and CD206 + macrophages, we report expression differences in diverse pathways and genes involved in energy metabolism, immune regulation and scavenging. This highlights a specialization of CD206- and CD206 + macrophages in islets and exocrine tissue.

Next, global gene expression was compared between islet CD206- and exocrine CD206- macrophages, and between islet CD206 + and exocrine CD206 + macrophages. The differences in these comparisons must be interpreted with caution, since there was differential expression in several genes and pathways that are highly expressed in parenchymal cells in the origin tissues. This suggests a contamination by neighboring parenchymal cell RNA. Due to the high sorting purity shown by both flow cytometry and MuSiC, this was a surprising result. While low levels of contaminating parenchymal cells could lead to transcriptomic differences due to the entirely different cell compositions and high rates of transcription of some genes in islet- and exocrine parenchyma, e.g. insulin, glucagon, amylase, chymotrypsin and lipase, in our previous study of pancreatic microvascular endothelial cells with almost identical methodology we did not find differences of parenchymal origin in the RNA-Seq analyses^36^. Macrophages are characterized by their phagocytic capabilities and are known to phagocytose damaged parenchymal cell contents in their vicinity. This has been reported previously in sorted rat-islet myeloid cells, and was suggested to be an effect of the islet isolation procedures^37^. In the cancer setting, circulating macrophage-like cells have been shown to contain internalized tumor tissue-expressed proteins^38^, suggesting that similar processes can occur in vivo.

Alternatively, macrophages could take up material from neighboring intact cells. Murine islet macrophages monitor their environment with filopodia and are capable of engulfing insulin-containing granules from intact neighboring beta-cells^39^. Further, cultured human islets have been shown to produce exosomes containing mRNA for insulin and beta-cell identity markers^40^, and macrophages have been shown to have an important general function in the clearance of exosomes^41^. Further, myeloid cells have previously been shown to be capable of taking up naked^42^ and circular RNA^43^. Thus, the parenchymal RNA measured in our samples could be due to macrophage uptake of parenchymal cell contents.

There are some additional limitations in our study. Since macrophages are highly heterogeneous, there are likely additional subpopulations present within the analyzed CD206 + and CD206- populations. A recent study of exocrine human pancreatic tissue using Iba1 to stain macrophages has shown the presence of macrophages with CD206 + CD163 + HLA-DR- or triple-negative surface marker pattern^44^, and we did not sort such macrophages. The islet isolation procedures could affect macrophage transcriptomes, but analysis of macrophage global gene expression in situ is not currently practically feasible. For a more complete characterization of macrophages it would also have been preferable to include more donors with IGM, type 2 diabetes and study donors with type 1 diabetes or other pancreatic disease, but this was unfortunately not possible due to the scarcity of human pancreatic tissue.

Enrichments in neurogenesis-related sets were also likely due to expression in endocrine cells, which express neural growth factors such as *NTRK1* (also known as *TRKA)*, *NGF*^45^ and *NCAM1* (also known as *NCAM*)^46^. There were enrichments in general cellular processes, including cell signaling, which likely also reflect expression differences between parenchymal cells. Thus, we focus our discussion on the remaining gene sets.

In CD206- comparisons, two immune-regulation associated gene sets were enriched in islet samples. The involved molecules butyrophilins^47^ and PD-1^48^ are negative regulators of T-cell responses. Interestingly, treatment with PD-1 inhibition can cause type 1 diabetes and diabetic ketoacidosis as a rare side effect^49^. Whether intraislet CD206- macrophages contribute to islet immune homeostasis under normal conditions through mechanisms involving these pathways could be an interesting question for future research. Exocrine samples had enrichment in gene sets involved in diverse functions, including several proinflammatory gene sets. In short, comparisons between CD206- macrophages suggest a slightly different immunophenotype in islets compared with exocrine tissue, with enrichment in two anti-inflammatory gene sets in islets and multiple pro-inflammatory gene sets in exocrine tissue. This would need to be confirmed through further functional studies.

Similarly, in comparisons between CD206 + samples, two immune-related gene set were enriched in islets. None of the individual genes in the two sets PID_IL_12_STAT4_PATHWAY or PID_TCR-CALCIUM_PATHWAY were differentially expressed between CD206 + samples. In the comparisons between CD206- and CD206+, the prior set was enriched in CD206 + and interpreted as IL2RA-related. The PID_TCR_CALCIUM_PATHWAY contains some overlapping genes with this set, including IL2RA. The difference between CD206 + samples could reflect slightly higher activity in IL2RA-related processes in islet CD206 + than in exocrine CD206+.

In exocrine CD206 + eight sets had more apparent relevance in macrophages. These sets were involved in processes related to alpha-defensins, extracellular matrix, and immune activation and most were also enriched in exocrine CD206- compared with islet CD206-; these sets may reflect general differences between islet- and exocrine tissue macrophages.

Comparing samples from normoglycemic donors with samples from donors with intermediately elevated HbA1c or type 2 diabetes identified significant differences in a sparse number of genes and no gene set enrichments. This could be related to low power or to potential heterogeneity in donor characteristics (such as duration of disease, treatment and other individual factors).

In summary, we present global gene expression profiles from two distinct macrophage subsets in human pancreatic islets and exocrine tissue. Comparatively, pancreatic CD206 + macrophages showed enrichment in gene sets associated with complement, scavenging, and IL-10 and IL-2RA associated immunosuppression, while CD206- macrophages showed enrichment in gene sets associated with glycolysis, pro-inflammation and SPP1. A few potentially important differences in immune regulatory pathways were differentially enriched between islet- and exocrine macrophages. This work offers a platform for further study of human pancreatic macrophages in health and disease.

## Methods

### Ethics statement

Prior written informed consent for organ donation (for use in research) was obtained via online database (https://www.socialstyrelsen.se/en/apply-and-register/register/), if available. Alternatively, if no prior consent was available, informed consent was obtained verbally from the deceased’s next of kin by the attending physician and documented in the medical records of the deceased in accordance with Swedish law and as approved by the Regional Ethics Committee in Uppsala (Dnr 2023-01845-01). The present study protocol was approved by the Regional Ethics Committee in Uppsala (Dnr 2015/444). All experiments were carried out in accordance with applicable regulations and ethical guidelines. No organs were procured from prisoners.

### Human pancreatic tissue

Transplant-grade pancreatic tissue was acquired through the Nordic Network for Islet Transplantation at the in-house islet isolation facility at Rudbeck laboratory, Uppsala, Sweden. Islets and exocrine tissue were isolated using a previously described method^50^. Normoglycemic donors that yielded an islet fraction with islet purity > 85% were included in the study. Donors with elevated HbA1c or type 2 diabetes were included regardless of islet purity (median islet purity 86%, range 65–95%). In total nine normoglycemic donors, two donors with intermediately elevated HbA1c (HbA1c 42-48mmol/mol and three donors with type 2 diabetes were included. From each of these donors, 20,000 islet equivalents and a similar amount of exocrine tissue was collected and processed within 1 h after isolation finish.

Additionally, one donor with type 1 diabetes was included in the study. 100 islets were handpicked in this donor. This donor was excluded from the transcriptomic analyses. Donor details are provided in Supplementary Table S14.

### Single-cell dissociation and staining

Islet- and exocrine cell suspensions were washed in PBS followed by dissociation in 10 mL of Accutase for 20 min at room temperature. Cell clusters were frequently mechanically disaggregated with a P1000 pipette during dissociation. Cells were passed through a 40 μm filter, collected in CMRL-1066 medium supplemented with human serum (10%) and DNase I (100 units/mL), and then washed in washing buffer (PBS with 2.5% FCS and 1mM EDTA). Cells were incubated with anti-CD64 antibody at 4 °C for 10 min. FC-receptor blocking agent was added at 4 °C for 10 min. Cells were incubated with anti-CD45, anti-HLA-DR and anti-CD206 antibodies at 4 °C for 30 min followed by two washes. Cells were then passed through a 20 μm-filter. To evaluate pre-sorting CD206 expression and estimate total cell number, small aliquots of input samples were analyzed using a BD FACSVerse instrument. The remaining cells were resuspended in Tyto Running Buffer and loaded into a MacsQuant Tyto cartridge at an estimated concentration of 800,000 cells/mL. FACS data were analyzed using FlowJo software. Reagent details are listed in Supplementary Table S15.

### Cell sorting and FACS analysis

Macrophages were identified by low-to-medium side scatter and positive staining for HLA-DR, CD45, and CD64. For each donor, a small sample of islet cells was analyzed using a BD FACSVerse instrument to determine macrophage contents prior to sorting.

Macrophage subgroups were sorted sequentially using a MacsQuant Tyto instrument (a system using microchip-based flow cytometry with a mechanical valve sorting mechanism within a cartridge). First, CD206- macrophages were sorted from the islet- or exocrine sample, followed by reloading of the flow-through material and sorting of the CD206 + macrophage population. Starting tissue was alternated between donors. Cells passed through a 20 μm-filter within the sorting cartridge. The blue channel (HLA-DR staining) was used as trigger channel; the purple channel (CD45 staining) was used as speed channel. Samples were sorted at 8 °C and were then stored at 4 °C while the rest of the samples from the same donor were sorted. After all samples from one donor had been sorted, post-sorting analyses were performed using the BD FACSVerse instrument, using some cells (roughly 25% per sample). The gating strategy used in pre- and postsorting analysis of macrophage content and phenotype is shown in supplementary figure S9. The remaining cells were lysed in buffer RLT+ (a component of the Qiagen Allprep DNA/RNA Micro kit), homogenized using QIAshredder spin-columns and stored at -70 °C prior to RNA extraction.

### RNA extraction and library preparation

RNA was extracted using Qiagen Allprep DNA/RNA Micro kit according to the manufacturer’s instructions.

### Transcriptome sequencing and read processing

Library generation and sequencing was performed at NGI Stockholm (National Genomics Infrastructure facility, Stockholm node). Libraries were generated using the SMARTer Stranded Total RNA-Seq Kit v3 - Pico Input Mammalian kit. Libraries were sequenced using a NovaSeq 6000 instrument. Samples yielded a median of 20.3 million reads (range 11–28.7 million).

Read alignment and quantification was performed at NGI Stockholm, using the Nextflow-based nf-core/RNAseq pipeline^51^. The details of these procedures are described briefly in Supplementary Methods 1.

### Count processing, RNA-seq analysis, and data visualization

Salmon transcript abundance estimates were converted to estimated raw counts using DeSeq2^52^. The estimated counts were rounded to the closest integer values. Estimated counts were analyzed using R (version 4.12) in RStudio.

For the differential gene expression and gene set enrichment analyses, counts were processed using the default methods in the edgeR^53^ package. In short, low-expressed transcripts are filtered out using the filterByExpr function with at least 10 counts per sample in a minimum number of samples, and sample-wise correction factors are assigned by means of TMM normalization using the calcNormFactors function^54^.

For comparisons between macrophage subgroups within and between tissues, glycemic status was not considered during TMM-filtering. The smallest group thus contained 14 samples. 14,266 transcripts remained after filtering. For dispersion estimation and fitting of the generalized linear model, the paired nature of the samples and a combination of tissue origin and phenotype (CD206- or CD206+) were included in the design matrix.

For comparisons between glycemic states a separate filtering step and generalized linear model (glm)-fitting was used, in order to not filter out differentially expressed transcripts with expression in only one group. This filtering differed in size of the smallest group, since 2 donors with impaired glucose metabolism constituted the smallest group. 17,321 transcripts remained after this filtering step. Dispersion estimation and fitting of this glm was done without pairing between samples. This model was only used for the differential gene expression and CAMERA analyses between glycemic states (impaired glucose metabolism, type 2 diabetes and normoglycemia).

Principal component analysis was performed using the default settings in the R-package “PCAtools”^55^.

Differential gene expression analyses were performed using the edgeR-function “glmTreat” with the default log2 fold-change threshold of 1.2.

Gene set enrichment analysis was performed using CAMERA^56^ and the curated “C2: CP (Canonical pathways)” gene set collection, version 2023.1.Hs containing 3,090 gene sets, from MSigDB^57^).

Enriched gene sets were manually annotated into categories by the authors and visualized using the R package “ComplexHeatmap”^58^.

In order to estimate the cellular composition in our samples, Multi-subject Single Cell Deconvolution (MuSiC) analysis^59^ was performed using the MuSiC R package and the single-cell RNA sequencing data and annotations from Tosti et al.^60^. In MuSiC analysis, a single cell RNA-sequencing data set is used to ascribe a weight associated with each cell type for each transcript. The resulting matrix can then be used to estimate the cell type composition in a bulk RNA-sequencing data set. Before MuSiC analysis was performed, GENCODE annotations were trimmed to base ensemble gene IDs, which were then converted to gene symbols using BioMart^61^. Transcripts lacking gene symbols were removed. Duplicate gene symbols were removed, with only the most expressed transcript retained. 13,608 transcripts were retained for the MuSiC analysis.

As an initial quality control measure, the lists of differentially expressed genes between CD206- and CD206 + samples were analyzed for the sorting markers, previously reported M2 markers (as reported in^62,63^), monocyte markers^64^, and neutrophil markers^65^.

Data were visualized using GraphPad Prism (v 9.3.1) and the R-packages “PCATools”, “MuSiC”, complexHeatmap and “ggplot2”.

## Electronic supplementary material

Below is the link to the electronic supplementary material.

Supplementary Material 1

Supplementary Material 2

## Data Availability

Raw transcriptomic data are available at GEO (GSE281600). Full tables of differentially expressed genes and CAMERA results used in the differential gene expression and gene set enrichment analyses are available as separate supplementary files (Supplementary Tables 4-7 and 12-13; Supplementary Tables 8-11). Additional data is available from the corresponding author (A.J) on reasonable request.
