# Supplementary material for: Transcriptomic characterization of human pancreatic CD206- and CD206 + macrophages
Source: Sci Rep. 2025 Apr 8;15:12037. doi: 10.1038/s41598-025-96313-y (PMC11978877; doi:10.1038/s41598-025-96313-y)
Supplement: Supplementary file 2 — Supplementary Material 2 [file 41598_2025_96313_MOESM2_ESM.pdf]

Transcriptomic characterization of CD206- and CD206+ macrophages in human pancreatic islets and exocrine tissue

Authors: Alexander Jonsson [1*], Olle Korsgren[1,2], Anders Hedin[1]

1 Department of Immunology, Genetics and Pathology, Uppsala University, Uppsala, Sweden
2 Institute of Medicine, University of Gothenburg, Gothenburg, Sweden
*Correspondence to alexander.jonsson@igp.uu.se

Keywords: Human pancreas, pancreatic macrophages, diabetes, transcriptomics, pancreatic islets

Supplementary figure S1.
Fraction of macrophages with correct CD206 phenotype after sorting.

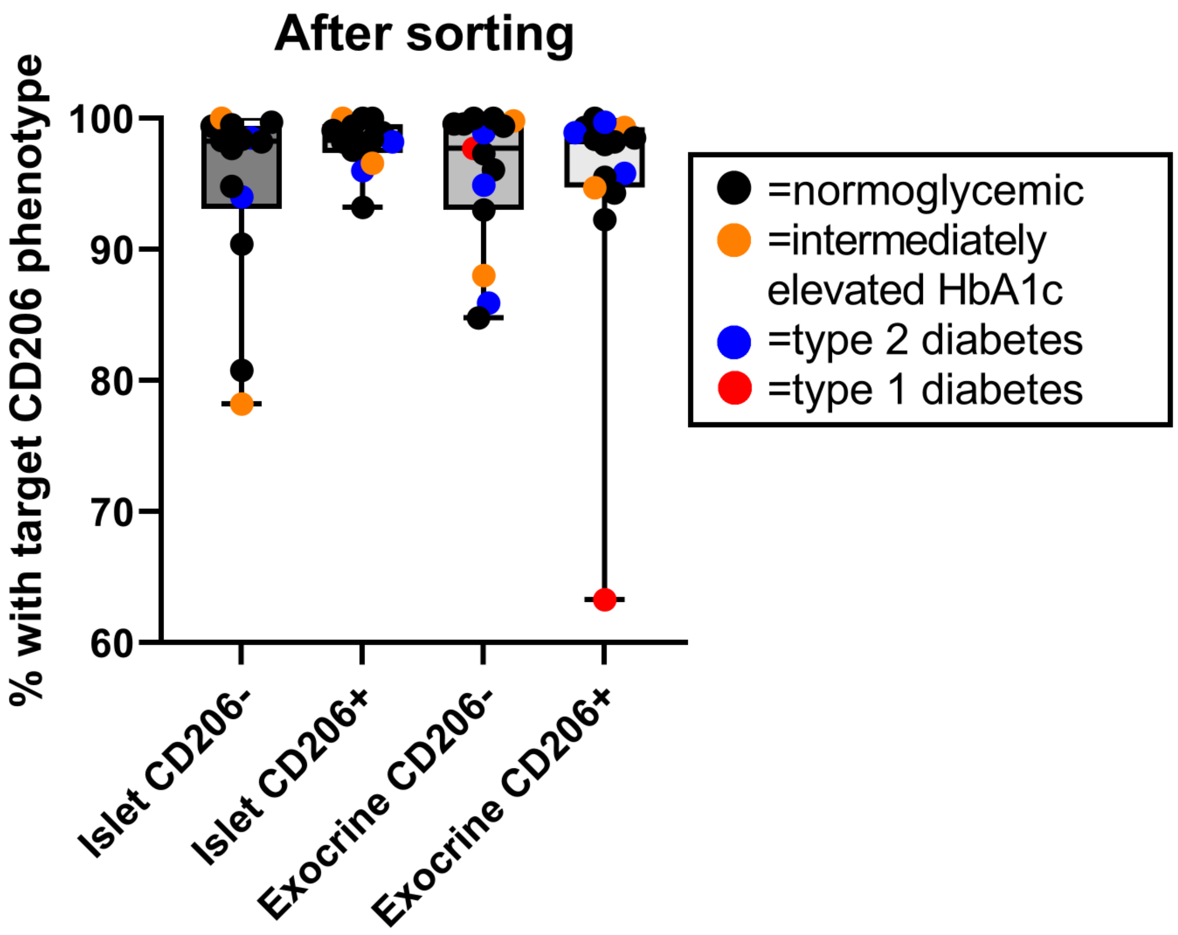

The fraction of macrophages with correct CD206 phenotype (out of the total number of macrophages) per sample. The samples from the donor with type 1 diabetes were excluded from further analyses for other reasons.

Supplementary figure S2. Cell surface expression of HLA-DR.

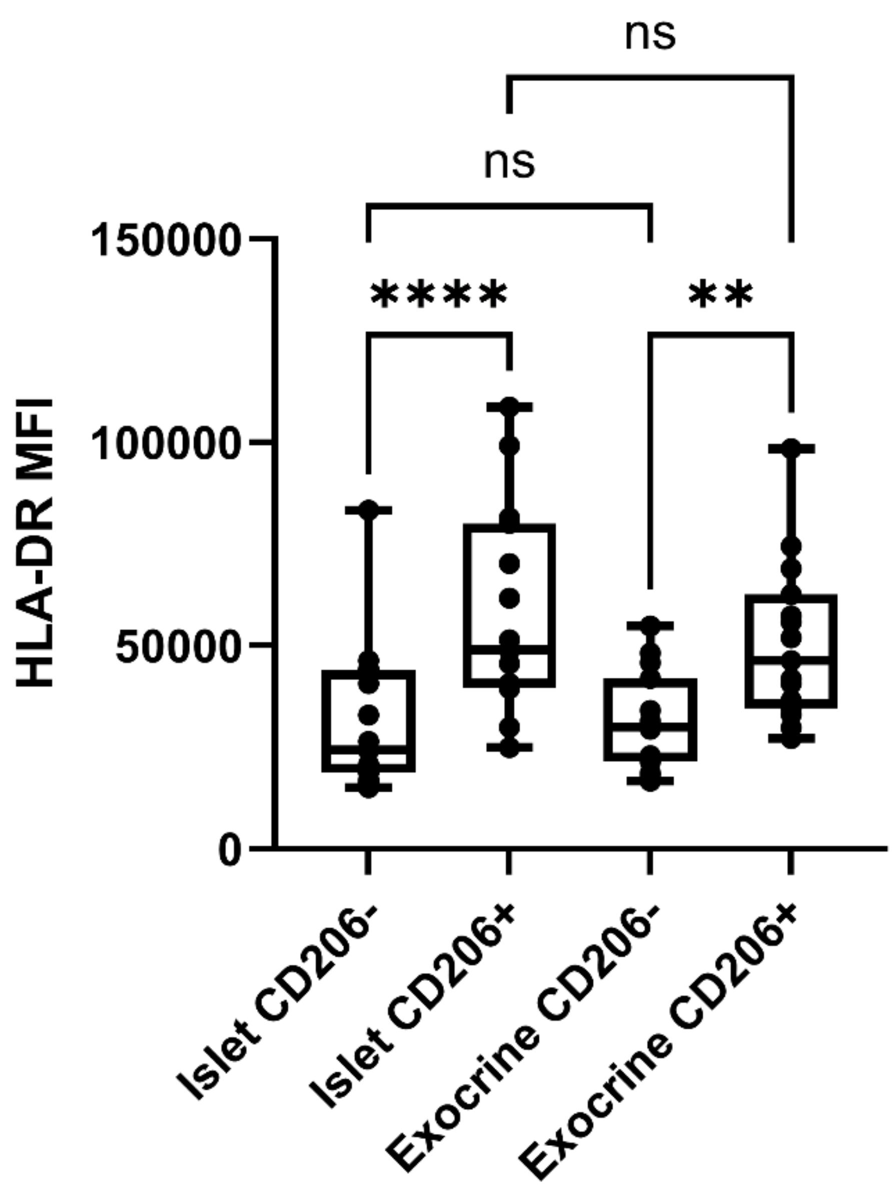

HLA-DR expression analyzed by FACS. MFI, mean fluorescence intensity. Each dot represents one sample. Statistical testing was performed using Friedman’s test followed by Dunn’s multiple comparisons test in GraphPad Prism. Multiplicity adjusted p-values are presented. ** = p <0.01, ****= p<0.0001 .

Supplementary figure S3. Correlations between donor data and macrophage content and phenotype.

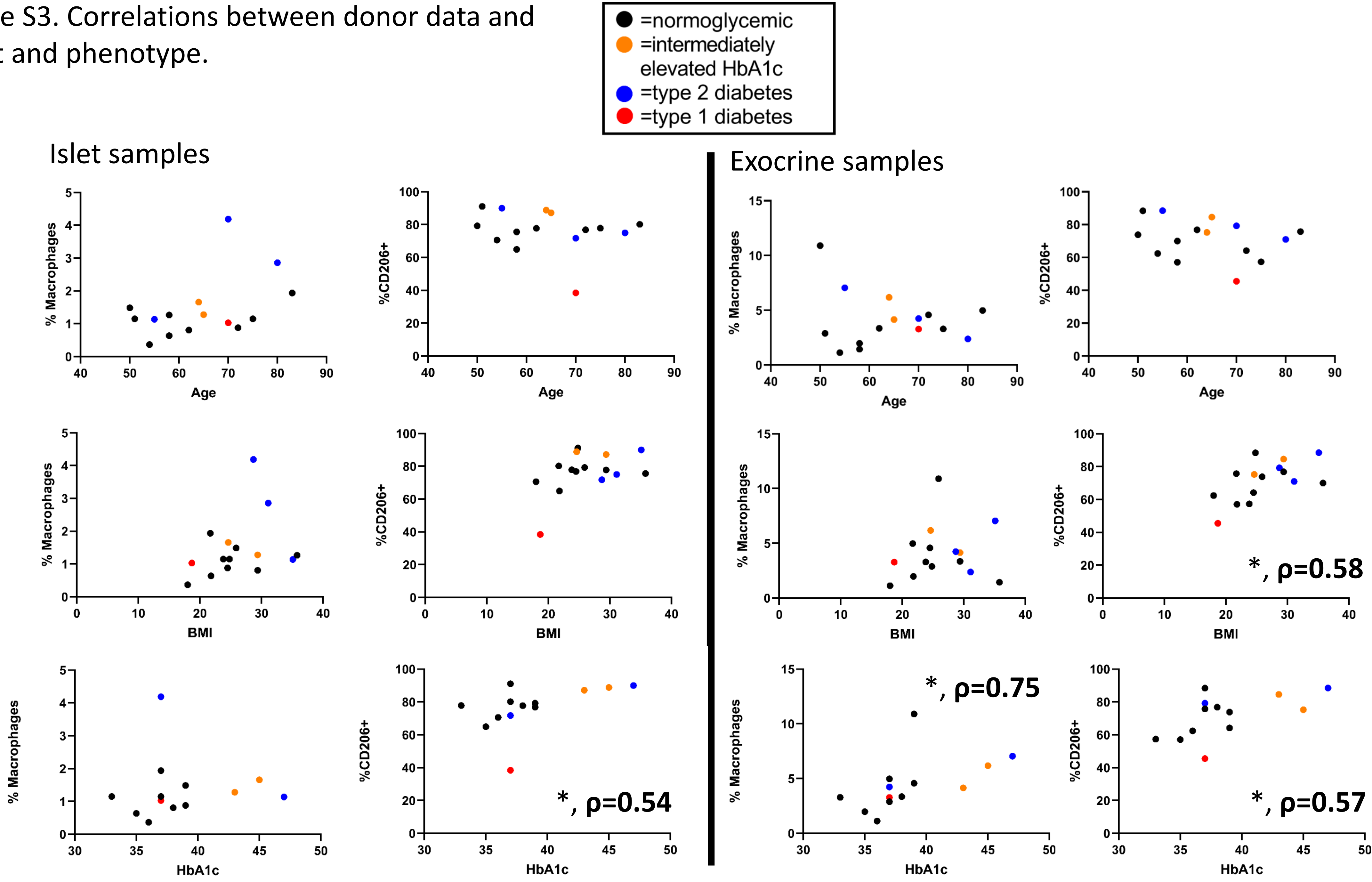

Correlations were analyzed using Spearman correlation analysis in GraphPad Prism. Spearman's ρ is reported for statistically significant (*=unadjusted p-value <0.05) correlations.

Supplementary figure S3 continued.

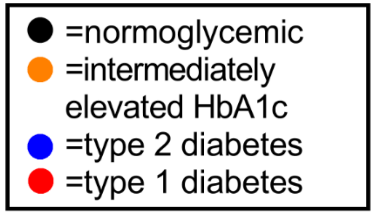

Correlation coefficient matrix

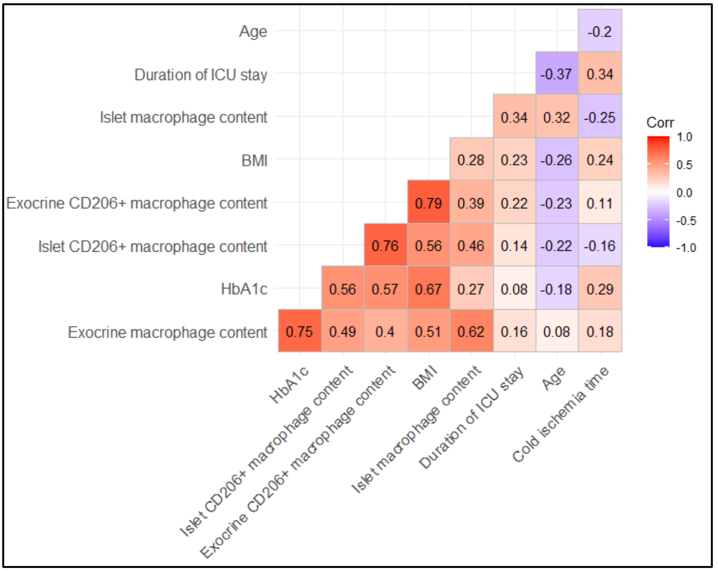

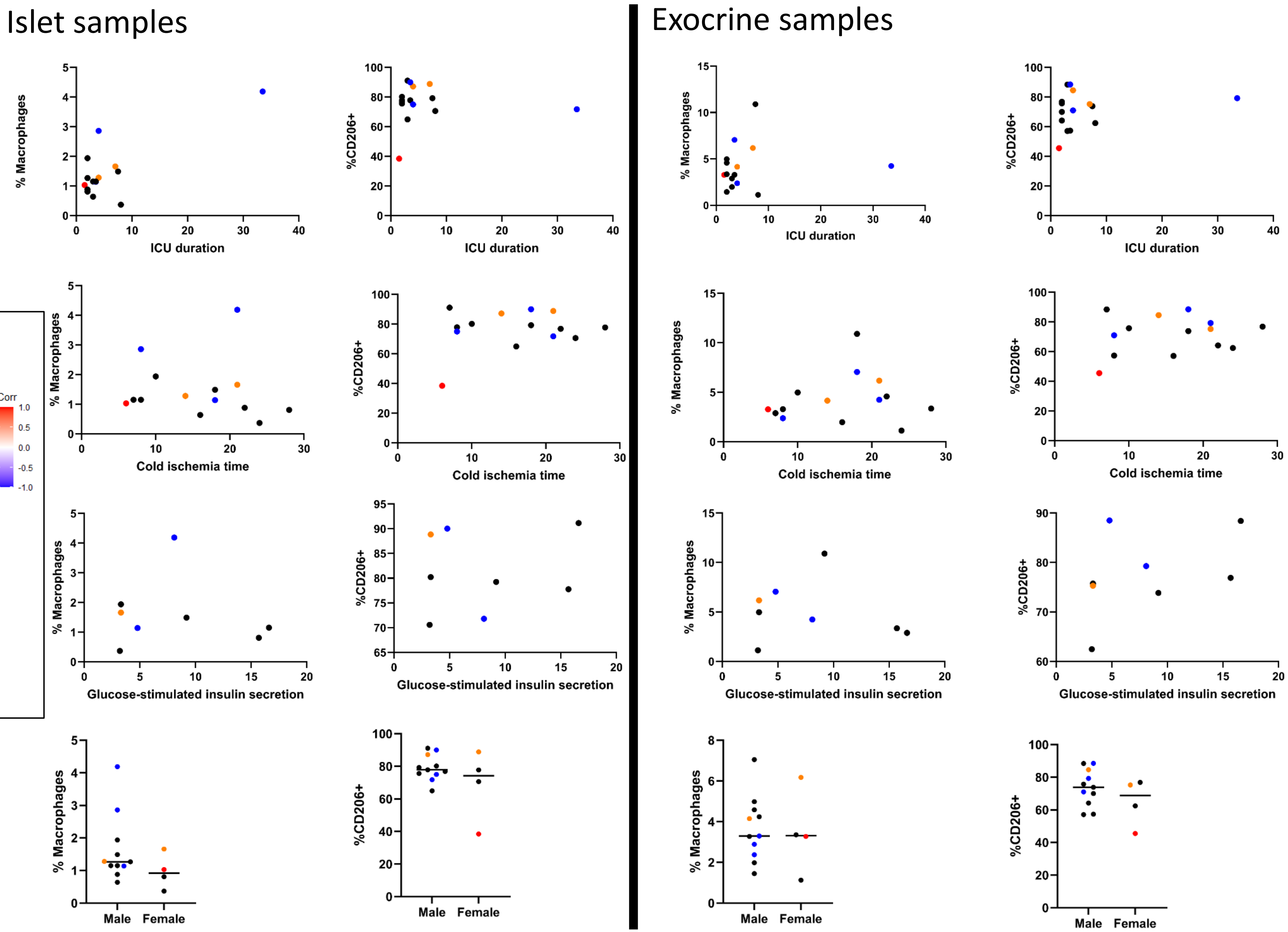

Differences between sexes were analyzed using Mann-Whitney test in GraphPad Prism.

Supplementary figure S4.

Gene expression of sorting markers and CD68

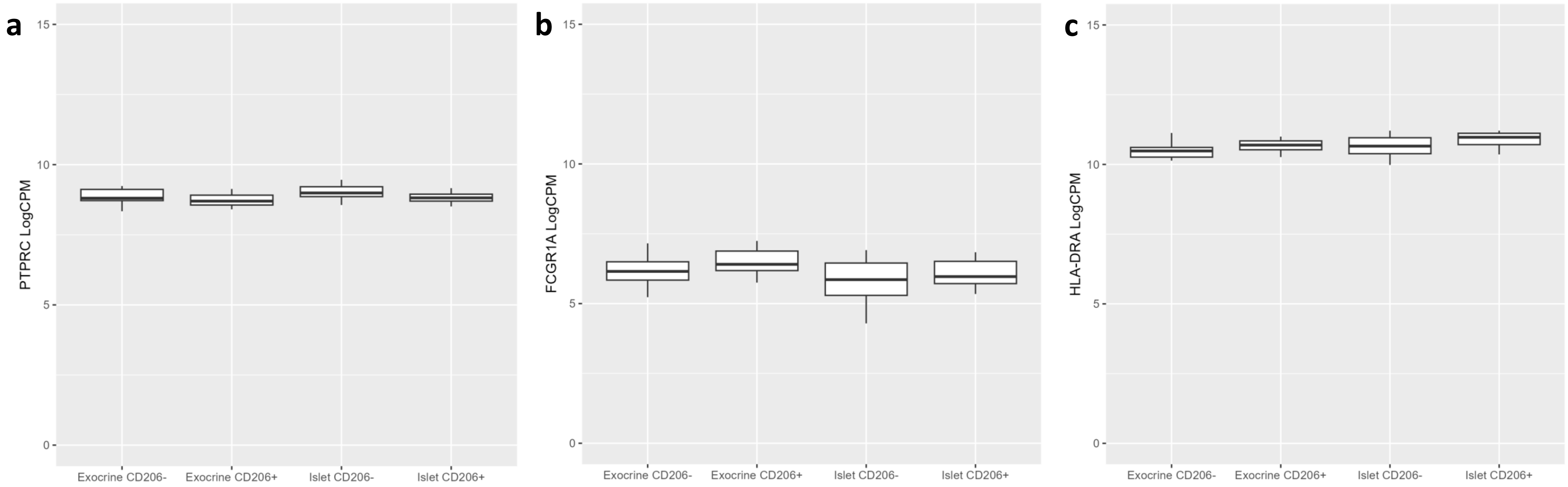

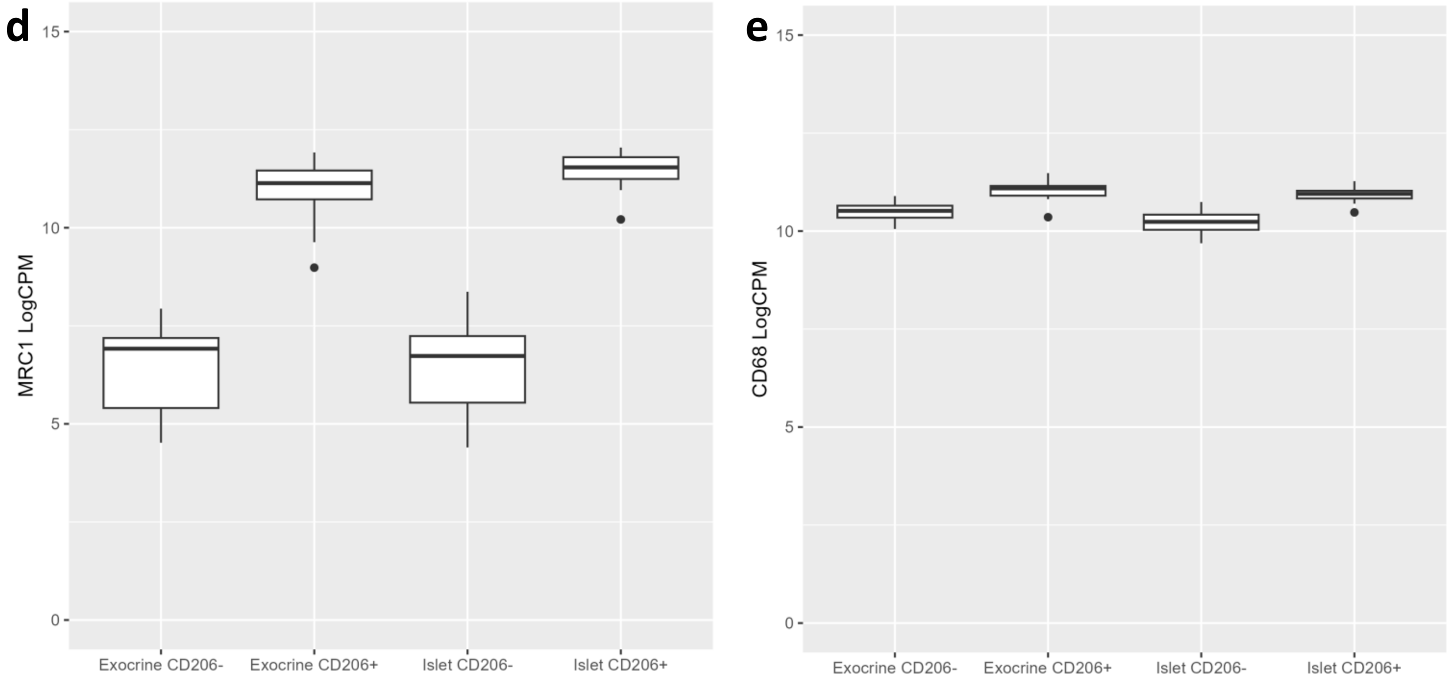

Log2-transformed counts per million for the genes for the sorting markers (a: CD45 (*PTPRC*), b: CD64 (*FCGR1A)*, c: HLA-DR (*HLA-DRA*) and d: CD206 (*MRC1*)) and e: CD68 plotted per group. Hinges represent 25th and 75th percentiles, whiskers represent range (up to a maximum of 1.5*interquartile range) and dots represent outliers.

Supplementary figure S5. Overlap between enriched gene set categories, islet CD206- macrophages.

Figure S5-S8: Functionally enriched gene sets (from the islet CD206- vs islet CD206+ and exocrine CD206- vs exocrine CD206+ analyses respectively) and the number of overlapping DEGs between sets are presented per macrophage subgroup. Significantly enriched genes (FDR<0.3) shared between gene sets are indicated by a connecting vertical line and the number of shared genes (intersection size) is depicted at the top of the graph.

Overlap analysis was performed and visualized using the R package “ComplexUpset”[1] and in this analysis all genes differentially expressed in the same direction as the gene sets with an FDR<0.3 were included.

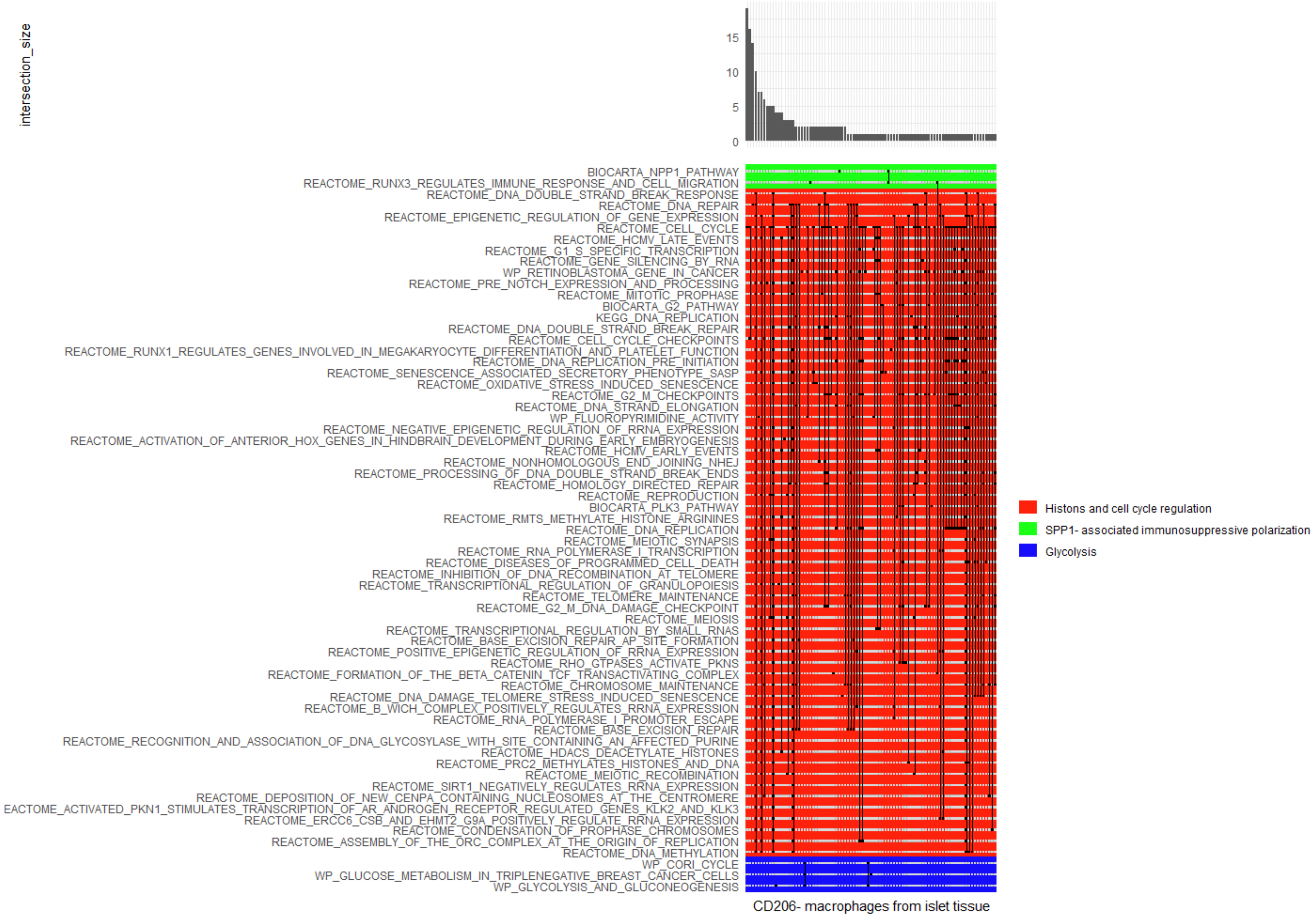

Supplementary figure S6. Overlap between enriched gene set categories, islet CD206+ macrophages.

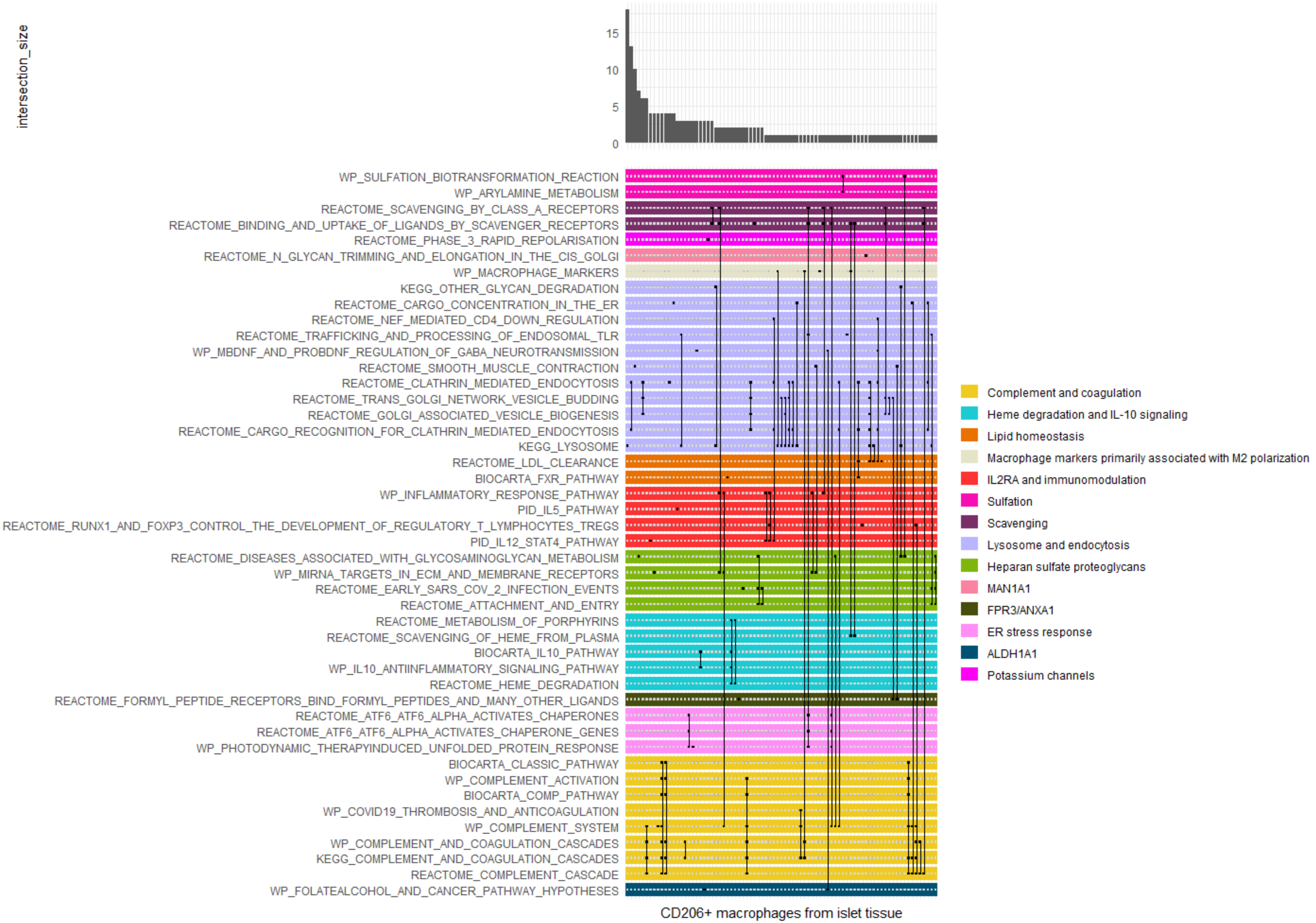

Functionally enriched gene sets (from the islet CD206- vs islet CD206+ and exocrine CD206- vs exocrine CD206+ analyses respectively) and the number of overlapping DEGs between sets are presented per macrophage subgroup. Significantly enriched genes (FDR<0.3) shared between gene sets are indicated by a connecting vertical line and the number of shared genes (intersection size) is depicted at the top of the graph.

Supplementary figure S7. Overlap between enriched gene set categories, exocrine CD206- macrophages.

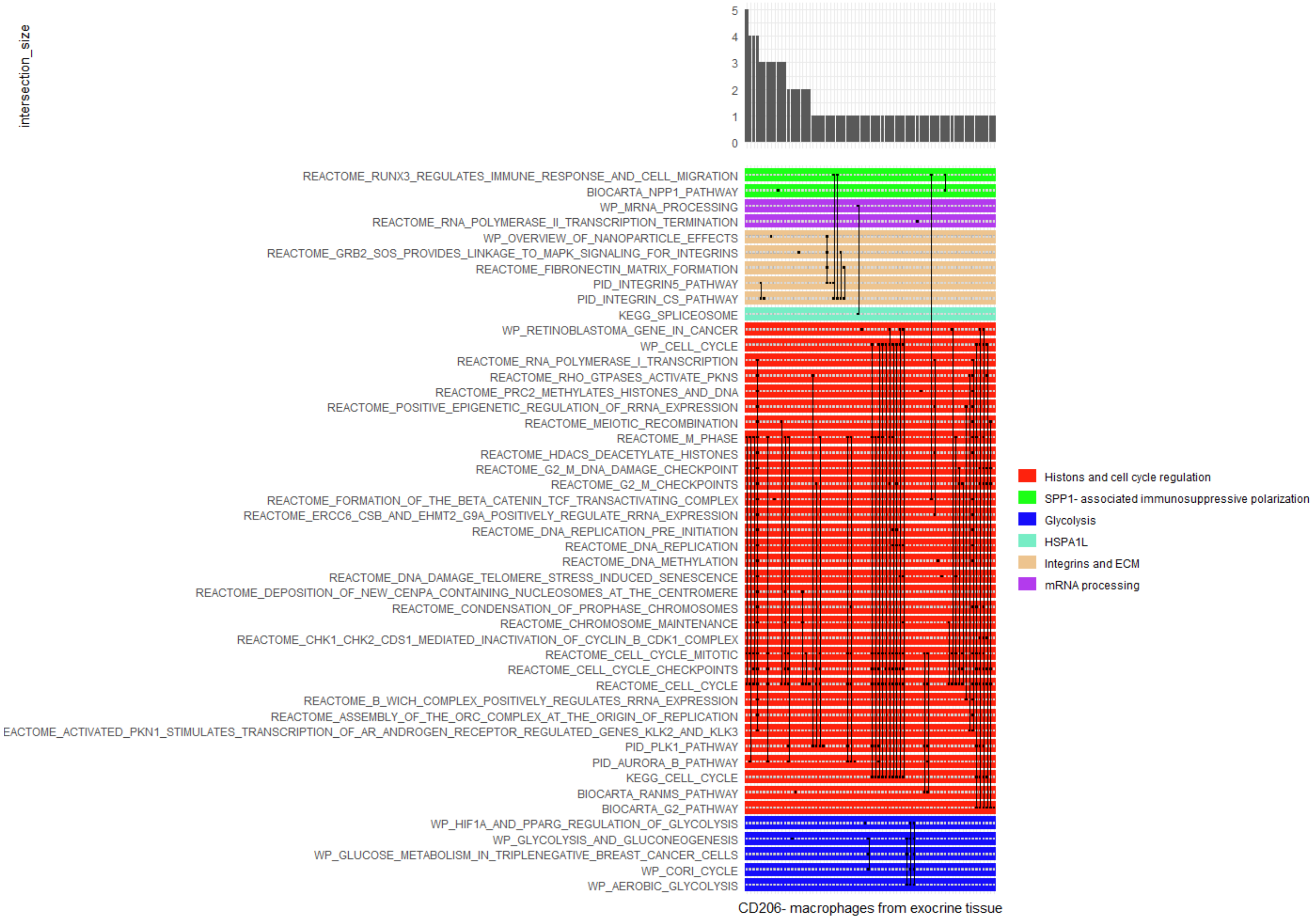

Functionally enriched gene sets (from the islet CD206- vs islet CD206+ and exocrine CD206- vs exocrine CD206+ analyses respectively) and the number of overlapping DEGs between sets are presented per macrophage subgroup. Significantly enriched genes (FDR<0.3) shared between gene sets are indicated by a connecting vertical line and the number of shared genes (intersection size) is depicted at the top of the graph.

Supplementary figure S8. Overlap between enriched gene set categories, exocrine CD206+ macrophages.

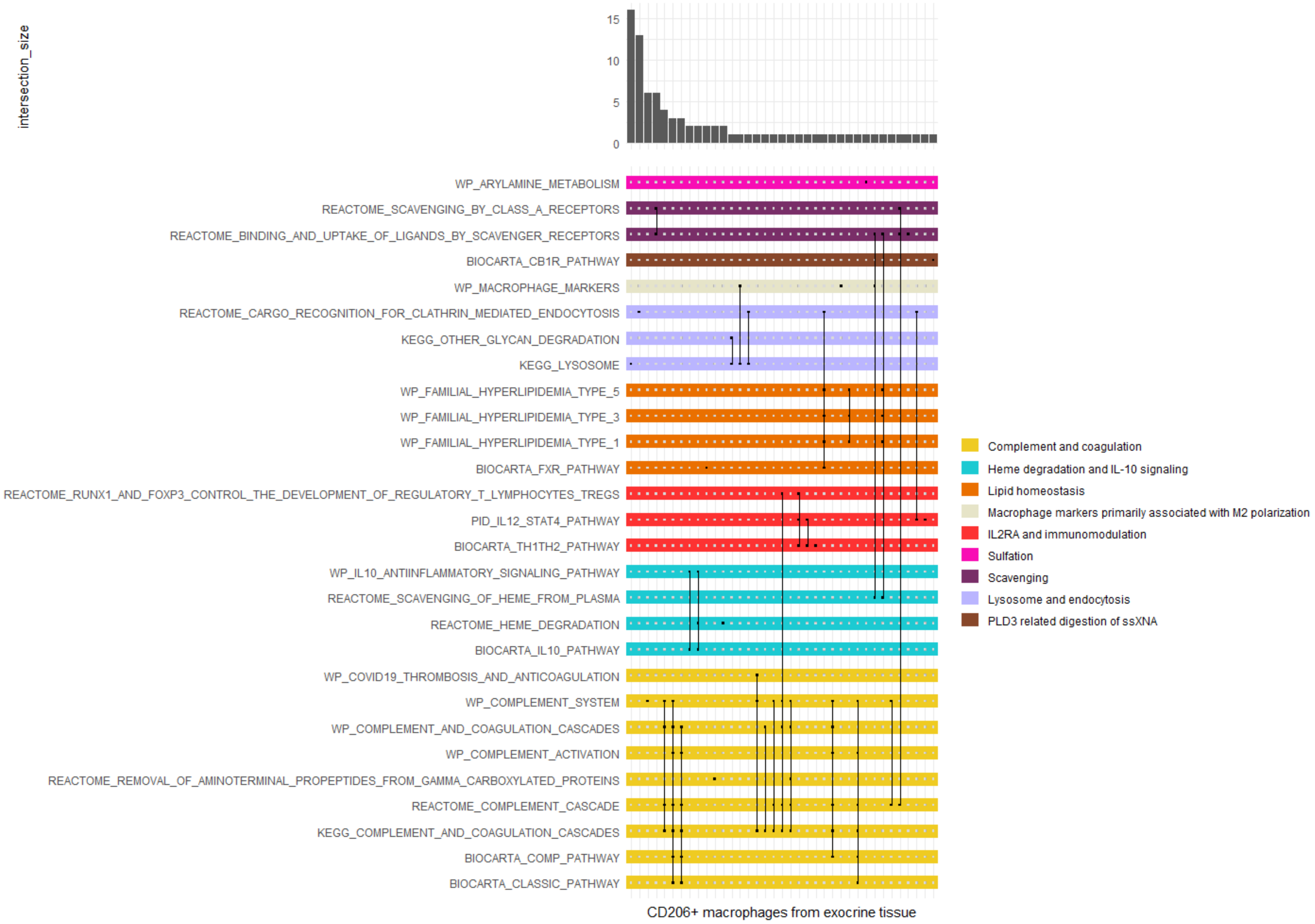

Functionally enriched gene sets (from the islet CD206- vs islet CD206+ and exocrine CD206- vs exocrine CD206+ analyses respectively) and the number of overlapping DEGs between sets are presented per macrophage subgroup. Significantly enriched genes (FDR<0.3) shared between gene sets are indicated by a connecting vertical line and the number of shared genes (intersection size) is depicted at the top of the graph.

Supplementary figure S9. Gating strategy for FACS-analysis.

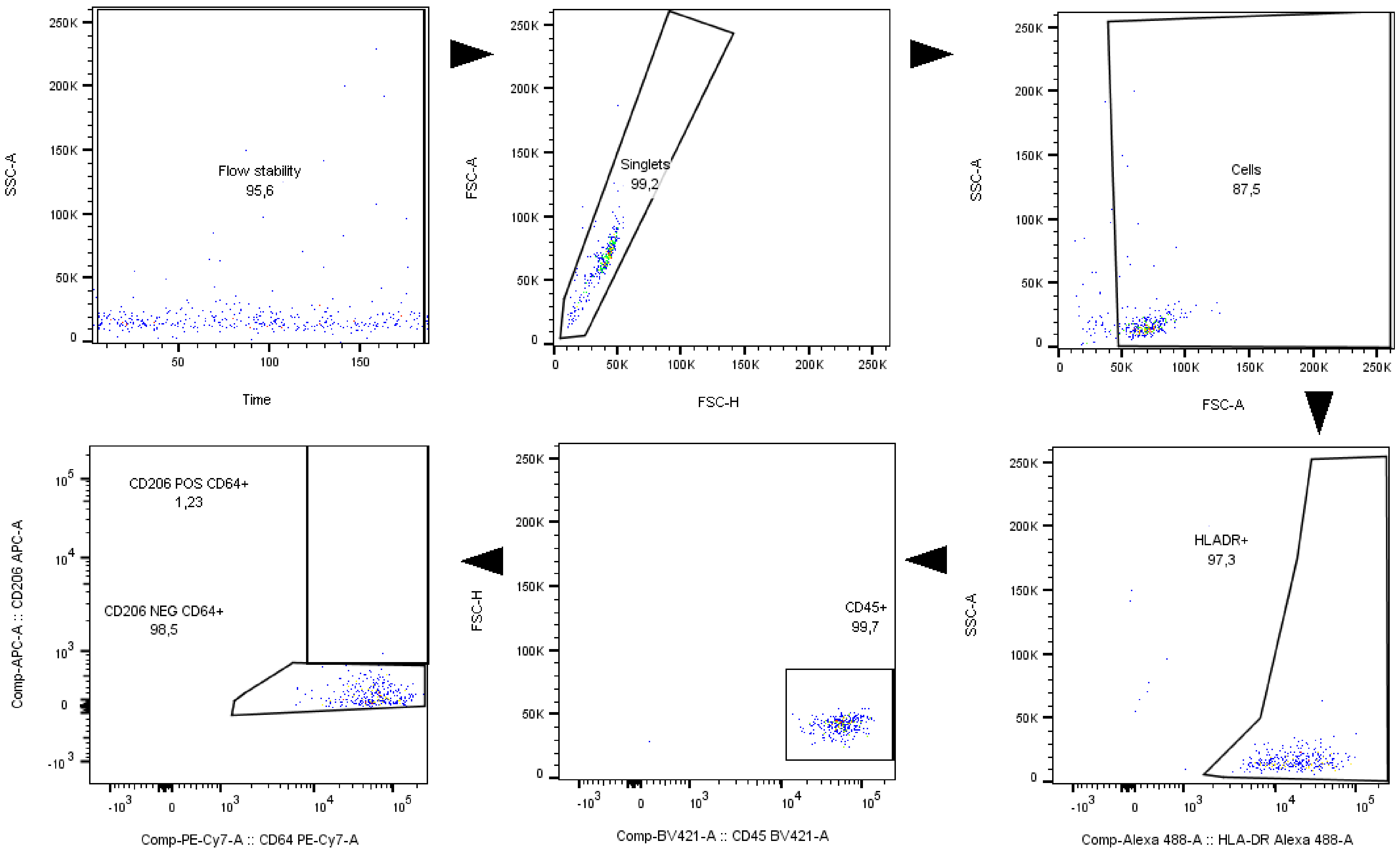

A representative sorted macrophage sample (islet CD206- sample of donor 7) illustrates the sequential gating strategy (arrowheads).

Supplementary table S1. Analysis of M2 markers in macrophage samples.

M2 Markers		
Gene	Exocrine CD206+ enrichment compared with exocrine CD206-?	Islet CD206+ enrichment compared with islet CD206-?
CD163	Yes	Yes
MSR1	Yes	Yes
CCL18	Yes	Yes
CD209	Yes	Yes
CLEC10A	Yes	Yes
IGF1	Yes	Yes
IL10	Yes	Yes
MERTK	Yes	Yes
CD86	No	No
TGFB	No	No
NOS2	No	No
ARG1	No	No
CLEC7A	No	No
VEGFA	No	No
IL1RN	No	Yes

Supplementary table S2. Analysis of monocyte markers in macrophage samples.

Monocyte markers			
Gene	Enrichment in exocrine macrophages?	Enrichment in islet macrophages?	Expressed in macrophages in human protein atlas?
APOBEC3A	CD206-	CD206-	Yes
CD14	CD206+	CD206+	Yes
CCR2	None	None	
S100A9	None	None	
S100A8	CD206-	None	Yes
CSF3R	CD206-	CD206-	Yes
CD7	None	None	
TET2	None	None	
CD40	None	None	
DYSF	None	None	
CMKLR1	CD206+	CD206+	Yes
MEFV	None	None	
HCK	None	None	
ITGAX	None	None	
SELE	None	None	
TLR4	CD206+	CD206+	Yes
AR	None	None	
CXCR4	None	None	
CD86	None	None	
TNFRSF14	None	None	
ADA2	None	None	
MGMT	None	None	
CD33	None	CD206+	Yes
ITGAM	None	CD206+	Yes
ACE	None	None	
FUT4	None	None	
SELL	None	None	
CD163	CD206+	CD206+	Yes
FCGR1A	None	None	
FCGR2G	None	None	
ACP5	CD206+	CD206+	Yes
MRC1	CD206+	CD206+	Yes
GBP1	None	None	
OAS1	None	None	
IRF7	None	None	
PLSCR1	None	None	
MX1	None	None	
IL1RN	None	CD206+	Yes
IFIT1	None	None	
IDO1	None	None	
IFIT3	None	None	
CXCL10	None	None	

Monocyte markers			
Gene	Enrichment in exocrine macrophages?	Enrichment in islet macrophages?	Expressed in macrophages in human protein atlas?
CXCL10	None	None	
S100A4	CD206-	CD206-	
CLEC7A	None	None	
MNDA	None	None	
MS4A6A	CD206+	CD206+	Yes
CLEC12A	None	None	
CD48	None	CD206-	Yes
PRTN3	None	None	
FCGR3A	None	None	
VCAN	CD206-	None	Yes
IFITM3	CD206+	CD206+	Yes
FN1	CD206-	CD206-	Yes
ADGRE1	CD206-	CD206-	Yes
CD44	None	None	
CSF1R	None	None	
CX3CR1	CD206-	CD206-	Yes
ITGAL	None	CD206-	Yes
PECAM1	None	None	
PTPRC	None	None	
SPN	CD206-	CD206-	Yes
PSAP	None	None	
SPN1	None	None	
RGS1	None	None	
FCGR3B	None	None	
PADI4	None	CD206-	Yes
GHSR	None	None	
**Sum of CD206- & CD206+ enriched**	**9&8**	**10&11**	

Supplementary table S3. Analysis of neutrophil markers in macrophage samples.

Enriched in neutrophils in 75-100% of tissues				Enriched in neutrophils in 50-75% of tissues			
Gene	Enrichment in exocrine macrophages?	Enrichment in islet macrophages?	Expressed in macrophages in human protein atlas?	Gene	Enrichment in exocrine macrophages?	Enrichment in islet macrophages?	Expressed in macrophages in human protein atlas?
APOBEC3A	CD206-	CD206-	Yes	ADGRG3	None	None	
AQP9	None	None		BCL2A1	None	CD206-	Yes
ARG1	None	None		CEACAM3	None	None	
CD177	None	None		CMTM2	None	None	
CLEC4D	None	None		FPR1	None	None	
CSF3R	CD206-	CD206-	Yes	HAL	None	None	
CXCR1	None	None		IL18RAP	None	None	
CXCR2	None	None		ITGAX	None	None	
FCAR	None	None		LILRA5	None	None	
FCGR3B	None	None		MEFV	None	None	
FFAR2	None	None		MMP9	None	None	
FPR2	None	None		PGLYRP1	None	None	
MCEMP1	None	None		S100A9	None	None	
MGAM	None	CD206-	Yes	S100P	None	None	
MMP25	None	None		SELL	None	None	
NFE2	None	None		SLC26A8	None	None	
PADI4	None	None	Yes	TREM1	None	None	
PROK2	None	None		TREML2	CD206-	CD206-	Yes
S100A12	None	None	Yes	Sum CD206- & CD206+	1&0	2&0	
S100A8	CD206-	None	Yes				
VNN2	CD206+	None	Yes				
Sum CD206- & CD206+	3&1	3&0					

Supplementary table S3 continued.

Enriched in neutrophils in 25-50% of tissues			
Gene	Enrichment in exocrine macrophages?	Enrichment in islet macrophages?	Expressed in macrophages in human protein atlas?
ADAM8	CD206-	CD206-	Yes
ADGRE3	None	None	Yes
CD300E	CD206-	CD206-	Yes
CEACAM4	None	None	
CLEC4E	CD206+	CD206+	Yes
CYP4F3	None	None	
FCGR2A	CD206+	CD206+	Yes
FCN1	CD206-	CD206-	Yes
FGR	None	CD206-	Yes
FOLR3	None	None	
FUT7	CD206-	CD206-	Yes
GCA	CD206+	None	Yes
GLT1D1	None	None	
HRH2	None	CD206+	Yes
ICAM3	CD206-	CD206-	Yes
IL1R2	None	None	
KCNJ15	None	None	
LILRB3	None	CD206+	Yes
LRG1	None	None	
MAP3K8	None	CD206+	Yes
MGAM2	None	None	
MMP8	None	None	
NCF2	None	CD206+	Yes
NLRP12	CD206-	CD206-	Yes
NLRP6	CD206+	CD206+	Yes
PADI2	CD206-	CD206-	Yes
RETN	None	None	
RIPOR2	CD206-	None	Yes
S1PR4	None	None	
SERPINA1	None	CD206-	Yes
SIRPB1	None	None	
SLC11A1	None	None	
SOCS3	None	None	
SORL1	CD206-	CD206-	Yes
TLR2	None	None	
TNFRSF10C	None	None	
TREML4	None	None	
ZDHHC19	None	None	
Sum CD206- &CD206+	9&4	10&8	
**Total sum of CD206- & CD206+ enriched neutrophil markers**	**13&5**	**15&8**	

Supplementary table S4-S11

Due to the large sizes of the data tables, these tables are provided as a single separate file.

Supplementary table S12. Differentially expressed genes in islet CD206- macrophages between donors with elevated HbA1C or Type 2 diabetes compared with normoglycemia.

Positive logFC= enrichmnt in donors with intermediately elevated HbA1c (top) or donors with type 2 diabetes (bottom)

**Islet CD206-: Donors with intermediately elevated HbA1C vs donors with normoglycemia**						
ensembl_gene_id	hgnc_symbol	logFC	unshrunk.logFC	logCPM	PValue	FDR
ENSG00000114742	WDR48	1.17689150886171	1.17758015609947	4.669633112252	1.63143885753261E-06	0.0102174802422946
ENSG00000178568	ERBB4	8.04522178895265	8.89676658873478	-0.244169836805351	1.20768790751642E-07	0.00209183622460919
ENSG00000181873	IBA57	1.47341040974523	1.47519417598388	3.55897931785951	5.35011855210096E-06	0.0231673508602352
ENSG00000237973	MTCO1P12	2.39157656396764	2.39172544244352	7.69884231966575	0.0000017696692296567	0.0102174802422946
**Islet CD206-: Donors with type 2 diabetes vs donors with normoglycemia**						
ENSG00000138035	PNPT1	1.61920615263528	1.62016577597676	4.6030561231235	9.65589934431889E-06	0.0430447737790361
ENSG00000139405	RITA1	2.57345307227898	2.58127999067773	2.25839680101178	0.0000015486383944673	0.013411982815284
ENSG00000184012	TMPRSS2	6.48158600427774	7.32881531498379	0.379516252312322	1.01327227228032E-07	0.00175508890281675
ENSG00000187642	PERM1	6.31869423915984	6.57669511410727	0.0024893983986329	0.0000099404823691556	0.0430447737790361

Supplementary table S13. Differentially expressed genes in exocrine CD206+ macrophages between donors with type 2 diabetes and normoglycemia.

Positive logFC= enrichment in donors with type 2 diabetes, negative logFC=enrichment in donors with normoglycemia

Exocrine CD206+: Donors with type 2 diabetes vs donors with normoglycemia						
ensembl_gene_id	hgnc_symbol	logFC	unshrunk.logFC	logCPM	PValue	FDR
ENSG00000011465	DCN	2.71252231416663	2.7203046160826	2.11406378529181	0.0000935395641082904	0.0476529055858735
ENSG00000019549	SNAI2	2.73178769732368	2.7734987518254	0.538820520905638	0.0000933488248568219	0.0476529055858735
ENSG00000050165	DKK3	3.57241759533835	3.63138739809047	0.985113097924166	0.000100192445714583	0.0495838100634942
ENSG00000105664	COMP	5.33293162253761	5.71202765240059	0.166311684281601	0.0000113936658091107	0.0125273264017637
ENSG00000106624	AEBP1	3.84540967800052	3.8587376585994	2.8201126224078	0.0000355440682544402	0.0251641761436602
ENSG00000108821	COL1A1	4.55865041260407	4.57550067055117	2.8985935998461	5.10696554283761E-06	0.00982863890749892
ENSG00000111341	MGP	2.48601945905535	2.4886625514863	4.15643302703077	0.0000414994663111766	0.0266226761472552
ENSG00000112936	C7	3.48355129524175	3.48529100344013	4.57921108184987	9.14912025718905E-06	0.012011264948069
ENSG00000113361	CDH6	5.40942293364522	5.79023663543344	0.16376976575881	9.70831414311912E-06	0.012011264948069
ENSG00000116962	NID1	2.6017773528602	2.60634649617625	3.22928979863107	0.0000190366588561378	0.0173544193709033
ENSG00000118523	CCN2	3.11380994462235	3.11989561994608	3.10034668031304	0.000076487642927229	0.0427368536497591
ENSG00000122786	CALD1	2.40698218301655	2.40813545308268	5.12863303535148	0.0000004732701919415	0.00384508882770214
ENSG00000130635	COL5A1	4.05050119230712	4.07835160344379	2.01004749489854	5.76546004549257E-06	0.00998635334479767
ENSG00000131711	MAP1B	3.26166195435216	3.26735877333246	4.4037113183856	0.0000363203281329892	0.0251641761436602
ENSG00000133110	POSTN	5.00907039202602	5.38624336975451	-0.372559990224587	0.0000249062645821859	0.0205429242299068
ENSG00000134121	CHL1	5.87604231239207	6.55996474589811	-0.476161954174233	8.03360648707818E-06	0.012011264948069
ENSG00000134539	KLRD1	6.03763377174391	6.72261228360796	-0.283755088742124	0.0000565090585976509	0.0349569072846397
ENSG00000134853	PDGFRA	4.2866051491613	4.30293215475751	2.59382812940301	9.48457272079137E-07	0.00410705710242068
ENSG00000139329	LUM	3.8547302860912	3.88846170269792	1.53815446184274	0.0000157416415739272	0.0152374333086068
ENSG00000142156	COL6A1	2.35758052264082	2.36264939935804	3.05738390752095	0.0000832934978420959	0.045085208628842
ENSG00000142173	COL6A2	2.8193288557035	2.82421284859605	3.27637704259293	0.0000236748045879821	0.0205035645134219
ENSG00000142871	CCN1	3.21681903093603	3.22117117353016	3.48601798442922	0.0000158347554734093	0.0152374333086068
ENSG00000159403	C1R	3.21823174671213	3.22365873496128	3.14820636068519	1.50936347622403E-06	0.00522679951338237
ENSG00000163359	COL6A3	3.74283914949658	3.74617325607603	3.96545696587319	0.0000115719197753143	0.0125273264017637
ENSG00000163453	IGFBP7	2.08478840716279	2.08641125069953	4.39496481737553	0.0000589601726930245	0.0352154879729613
ENSG00000164056	SPRY1	2.13214748430239	2.13799792404593	2.79839460379165	0.0000050848758291541	0.00982863890749892
ENSG00000168542	COL3A1	3.79272276920027	3.80857923680635	2.47361973695166	5.13971049246682E-07	0.00384508882770214
ENSG00000172403	SYNPO2	2.39774259134944	2.40707093580528	2.25508661254791	0.0000405002588734021	0.0266226761472552
ENSG00000174348	PODN	3.77259396345581	3.80700560565266	1.33750175704262	9.43319069104208E-06	0.012011264948069
ENSG00000182326	C1S	3.36600172146264	3.37029342097283	3.34925690930268	6.65970006530016E-07	0.00384508882770214
ENSG00000187498	COL4A1	2.94689238210447	2.95033959194344	4.48060497127094	0.0000612373670386031	0.0353564144825215
ENSG00000188783	PRELP	3.8087508212421	3.84452751759922	1.40272253381743	0.0000353138850965941	0.0251641761436602
ENSG00000198542	ITGBL1	3.7958451515195	3.85366676491059	0.68512288495983	0.0000344640853491564	0.0251641761436602
ENSG00000206052	DOK6	5.90606335280007	6.5902372769874	-0.329366846456105	3.69003832673745E-06	0.00913073626534562
ENSG00000259522		6.96743443445611	7.22862418499178	0.552418393968608	1.81056504129636E-06	0.00522679951338237

Supplementary table S14.
Organ donor data.

Donor	Age	Sex	BMI	IGM/T1D/T2D/N?	HbA1C (mmol/mol; %)	Cause of death	ICU (d)	CIT (h)	Isolation centre	Estimated islet purity (%)	SI GSIS	Notes
D1	65	M	29.4	IGM	43; 6.1	CA	4	14	Rudbeck laboratory	90	n/a	
D2	70	F	18.7	T1D	37; 5.5	ICH	1.5	6	Rudbeck laboratory	Handpicked	n/a	Excluded from transcriptomic analyses due to low purity in sorted islet macrophages
D3	51	M	24.8	N	37; 5.5	CA	3	7	Rudbeck laboratory	88	16.6	
D4	55	M	35.1	T2D	47; 6.5	CA	3.5	18	Rudbeck laboratory	95	4.8	
D5	75	M	23.8	N	33; 5.2	SAH	3.5	8	Rudbeck laboratory	97	n/a	
D6	70	M	28.7	T2D	37; 5.5	Stroke	33.5	21	Rudbeck laboratory	65	8.1	
D7	83	M	21.7	N	37; 5.5	TBI, SDH	2	10	Rudbeck laboratory	93	3.3	
D8	50	M	25.9	N	39; 5.7	CA	7.5	18	Rudbeck laboratory	98	9.2	
D9	64	F	24.6	IGM	45; 6.3	CA	7	21	Rudbeck laboratory	86	3.3	
D10	62	F	29.4	N	38; 5.6	SAH	2	28	Rudbeck laboratory	92	15.7	
D11	72	M	24.5	N	39; 5.7	CA	2	22	Rudbeck laboratory	94	n/a	
D12	58	M	21.8	N	35; 5.4	TBI	3	16	Rudbeck laboratory	99	n/a	
D13	58	M	35.8	N	n/a	TBI	2	n/a	Rudbeck laboratory	92	n/a	
D14	54	F	18	N	36; 5.4	Hydrocephalus	8	24	Rudbeck laboratory	95	3.2	
D15	80	M	31.1	T2D	n/a	ICH	4	8	Rudbeck laboratory	84	n/a	

Modified version of the suggested checklist in Hart, N.J., Powers, A.C. Use of human islets to understand islet biology and diabetes: progress, challenges and suggestions. Diabetologia 62, 212–222 (2019). https://doi.org/10.1007/s00125-018-4772-2

Abbreviations: IGM, impaired glucose metabolism. N, normoglycemia. T1D, type 1 diabetes. T2D, type 2 diabetes. ICU, duration of stay in intensive care. CIT, cold ischemia time. SI GSIS, stimulation index glucose-stimulated insulin secretion.
M, male. F, female. CA, cerebral anoxia after cardiac arrest. ICH, intracerebral hemorrhage. SAH, subarachnoid hemorrhage. SDH, subdural hematoma. TBI, traumatic brain injury. n/a, not available.

Supplementary table S15. Reagents and software tools.

Single-cell dissociation and cell sorting			
**Product**	**Vendor/source**	**Catalogue number**	**Details**
Accutase	Thermofisher	A1110501	
Anti-CD45 antibody	BD	563879	Fluorophore: BV421
Anti-CD64 antibody	Miltenyi Biotec	130-116-301	Fluorophore: PE-Vio 770
Anti-CD206 antibody	BD	550889	Fluorophore: APC
Anti-HLA-DR antibody	BD	564516	Fluorophore: BB515
FcR-blocking reagent	Miltenyi Biotec	130-059-901	
MACSQuant Tyto Cartridge	Miltenyi Biotec	130-106-088	
Tyto running buffer	Miltenyi Biotec	130-107-206	
BD FACSVerse instrument	BD	Link to BD website	
MACSQuant Tyto Instrument	Miltenyi Biotec	130-103-931	
**RNA-extraction**			
QIAShredder Spin Columns	Qiagen	79656	
Allprep DNA/RNA Micro kit	Qiagen	80284	
RNA-Seq			
SMARTer Stranded Total RNA-Seq Kit v3 - Pico Input Mammalian kit	Takara Bio	634487	
NovaSeq 6000 system	Illumina	Link to Illumina website	
**Data analysis**			
FlowJo	FlowJo LLC	Link to FlowJo website	Version 10.5.3
R	https://www.r-project.org/		Version 4.1.2
RStudio			Version 2023.3.0.386
DeSeq2	R-package		
edgeR	R-package		
PCATools	R-package		
MuSiC	R-package		
CAMERA	R-package		
ggplot2	R-package		
C2:CP gene set collection	MSigDB	Link to MSigDB archive	Version c2.cp.v2023.1.Hs.entrez.gmt

Supplementary methods

Read alignment and quantification.

FASTQ-files were merged and subsampled with fq. Strandness was inferred using Salmon[1] and quality checked with FastQC[2]. UMIs were removed using UMI-tools[3] and adapter and quality trimming was performed using Trim Galore[4]. Genome contaminants and ribosomal RNA were removed with BBSplit and SortMeRNA[5] respectively. Reads were then aligned using STAR[6] and quantified using Salmon[1]. In post-processing, SAMtools[7] was used to sort and index the reads, Picard MarkDuplicates was used to mark duplicate reads and BEDtools[8] genomecov was used to create bigWig coverage files. Transcript assembly and quantification was then carried out using StringTie[9]. Final QC was conducted using RSeQC[10], Preseq[11], Qualimap[12], dupRadar [13], DESeq2[14] and MultiQC[15].

Supplementary references

[1] Michał Krassowski, Arts M, Lagger C, Max. krassowski/complex-upset: v1.3.5 2022. https://doi.org/10.5281/ZENODO.3700590.

[2] Patro R, Duggal G, Love MI, Irizarry RA, Kingsford C. Salmon provides fast and bias-aware quantification of transcript expression. Nat Methods 2017;14:417–9. https://doi.org/10.1038/nmeth.4197.

[3] Andrews S. FastQC: a quality control tool for high throughput sequence data. 2010. Available online at: http://www.bioinformatics.babraham.ac.uk/projects/fastqc n.d.

[4] Smith T, Heger A, Sudbery I. UMI-tools: modeling sequencing errors in Unique Molecular Identifiers to improve quantification accuracy. Genome Res 2017;27:491–9. https://doi.org/10.1101/gr.209601.116.

[5] Krueger F. Trim Galore. https://www.bioinformatics.babraham.ac.uk/projects/trim_galore/

[6] Kopylova E, Noé L, Touzet H. SortMeRNA: fast and accurate filtering of ribosomal RNAs in metatranscriptomic data. Bioinformatics 2012;28:3211–7. https://doi.org/10.1093/bioinformatics/bts611.

[7] Dobin A, Davis CA, Schlesinger F, Drenkow J, Zaleski C, Jha S, et al. STAR: ultrafast universal RNA-seq aligner. Bioinformatics 2013;29:15–21. https://doi.org/10.1093/bioinformatics/bts635.

[8] Danecek P, Bonfield JK, Liddle J, Marshall J, Ohan V, Pollard MO, et al. Twelve years of SAMtools and BCFtools. GigaScience 2021;10:giab008. https://doi.org/10.1093/gigascience/giab008.

[9] Quinlan AR, Hall IM. BEDTools: a flexible suite of utilities for comparing genomic features. Bioinformatics 2010;26:841–2. https://doi.org/10.1093/bioinformatics/btq033.

[10] Kovaka S, Zimin AV, Pertea GM, Razaghi R, Salzberg SL, Pertea M. Transcriptome assembly from long-read RNA-seq alignments with StringTie2. Genome Biol 2019;20:278. https://doi.org/10.1186/s13059-019-1910-1.

[11] Wang L, Wang S, Li W. RSeQC: quality control of RNA-seq experiments. Bioinformatics 2012;28:2184–5. https://doi.org/10.1093/bioinformatics/bts356.

[12] Daley T, Smith AD. Predicting the molecular complexity of sequencing libraries. Nat Methods 2013;10:325–7. https://doi.org/10.1038/nmeth.2375.

[13] Okonechnikov K, Conesa A, García-Alcalde F. Qualimap 2: advanced multi-sample quality control for high-throughput sequencing data. Bioinformatics 2016;32:292–4. https://doi.org/10.1093/bioinformatics/btv566.

[14] Sayols S, Scherzinger D, Klein H. dupRadar: a Bioconductor package for the assessment of PCR artifacts in RNA-Seq data. BMC Bioinformatics 2016;17:428. https://doi.org/10.1186/s12859-016-1276-2.

[15] Love MI, Huber W, Anders S. Moderated estimation of fold change and dispersion for RNA-seq data with DESeq2. Genome Biol 2014;15:550. https://doi.org/10.1186/s13059-014-0550-8.

[16] Ewels P, Magnusson M, Lundin S, Käller M. MultiQC: summarize analysis results for multiple tools and samples in a single report. Bioinformatics 2016;32:3047–8. https://doi.org/10.1093/bioinformatics/btw354.
